# Supplementary material for: Direct neurotransmitter activation of voltage-gated potassium channels
Source: Nat Commun. 2018 May 10;9:1847. doi: 10.1038/s41467-018-04266-w (PMC5945843; doi:10.1038/s41467-018-04266-w)
Supplement: Supplementary file 1 — Supplementary Information [file 41467_2018_4266_MOESM1_ESM.pdf]

Direct neurotransmitter activation of voltage-gated potassium channels

Manville *et al.*

Supplementary Figures and Tables

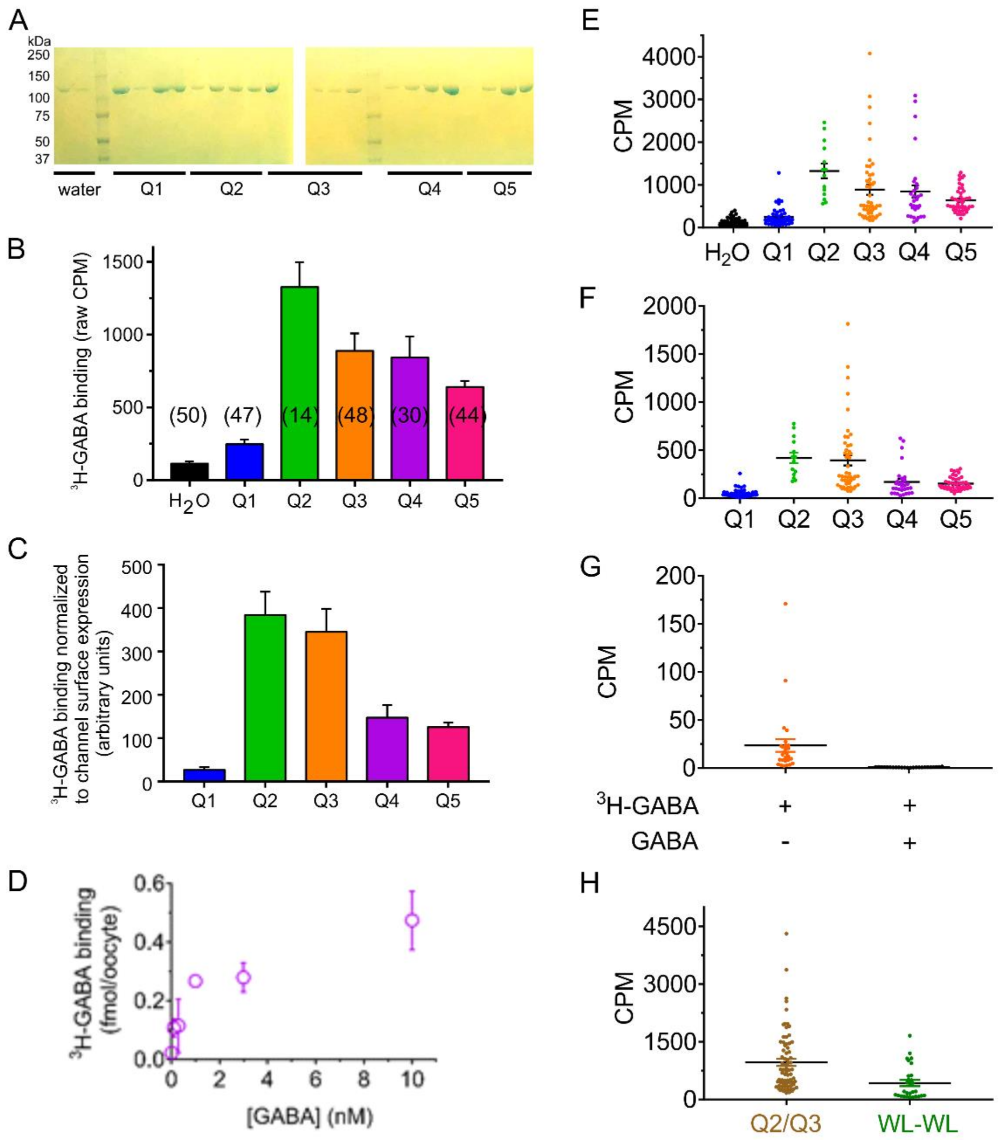

Supplementary Figure 1. Correction of 3H-GABA binding for channel protein surface expression

A. Representative Coomassie staining of surface-biotinylated proteins in water or KCNQ cRNA-injected oocytes (*n* = 4 lysates per group, each pooled from 5 oocytes). Duplicate gels showed the same pattern, ruling out gel-loading errors.

B. Mean raw scintillation CPMs for oocytes injected with water or channel cRNA as indicated; *n* values for each group in parentheses. Error bars indicate SEM.

C. Mean ^{3}H-GABA binding corrected for channel protein relative surface expression, and with mean water-injected CPM subtracted (*n* as in panel B). See Fig. 1 for statistical analysis. Error bars indicate SEM.

D. Expansion of lower [GABA] range points for KCNQ3 mean GABA saturation binding curve plot, all details as for main plot (Fig. 1j). Error bars indicate SEM.

E. Scatter plot corresponding to Supplementary Figure 1 B.

F. Scatter plot corresponding to Fig 1h/Supplementary Figure 1C.

G. Scatter plot corresponding to Figure 1i.

H. Scatter plot corresponding to Figure 3j.

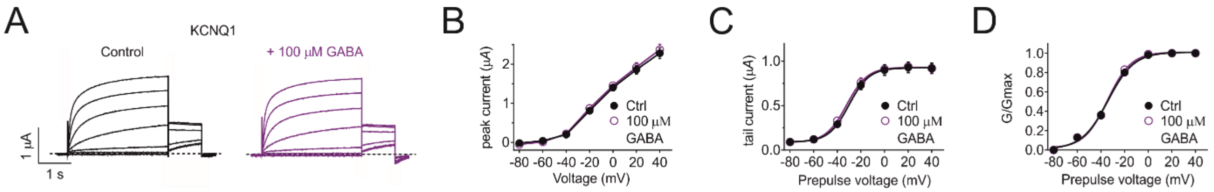

Supplementary Figure 2. Effects of GABA on KCNQ1 channels

A. Averaged KCNQ1 traces in the absence (black) and presence (purple) of GABA (100 μM), *n* = 4.
B. Mean peak current-voltage relationship for recordings as in panel A, *n* = 4. Error bars indicate SEM.
C. Mean tail current versus prepulse voltage relationships as in panel A, *n* = 4. Error bars indicate SEM.
D. Mean normalized tail current versus prepulse voltage relationships as in panel A, *n* = 4. Error bars indicate SEM.

**Q1**	**Normalized tail current $V_{0.5}$ (mV)**	**Non-normalized tail current $V_{0.5}$ (mV)**	**Slope (mV)**
**Ctrl**	-34.1 ± 0.9 (n =4)	-30.1 ± 2.2 (n =4)	10.9 ± 0.9 (n =4)
**100 μM GABA**	-34.2 ± 0.8 (n =4)	-32.6 ± 1.9 (n =4)	9.9 ± 0.7 (n =4)

Supplementary Table 1. Summary of Effects of GABA on KCNQ1 channels.

Values indicate mean ± SEM.

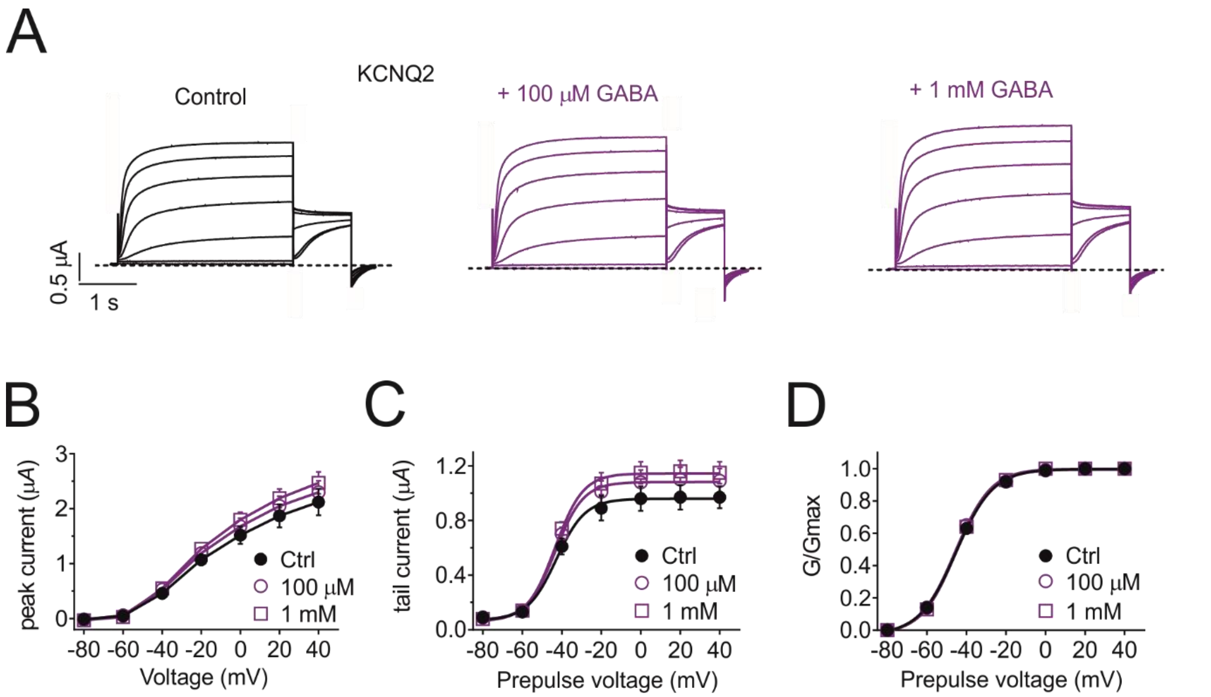

Supplementary Figure 3. KCNQ2 activity is relatively insensitive to GABA

A. Averaged KCNQ2 traces in the absence (black) and presence (purple) of GABA (100 μM; middle, 1 mM; right), *n* = 6.
B. Mean peak current-voltage relationship for recordings as in panel A, *n* = 6. Error bars indicate SEM.
C. Mean tail current versus prepulse voltage relationships as in panel A, *n* = 6. Error bars indicate SEM.
D. Mean normalized tail current versus prepulse voltage relationships as in panel A, *n* = 6. Error bars indicate SEM.

**Q2**	**Normalized tail current $V_{0.5}$ (mV)**	**Non-normalized tail current $V_{0.5}$ (mV)**	**Slope (mV)**
**Ctrl**	-45.2 ± 0.6 (n =6)	-42.8 ± 2.8 (n =6)	9.3 ± 0.6 (n =6)
**100 μM GABA**	-45.8 ± 0.4 (n =6)	-43.2 ± 2.5 (n =6)	9.0 ± 0.3 (n =6)
**1 mM GABA**	-45.1 ± 0.4 (n =6)	-43.4 ± 2.3 (n =6)	8.7 ± 0.3 (n =6)

Supplementary Table 2. Summary of Effects of GABA on KCNQ2 channels.

Values indicate mean ± SEM.

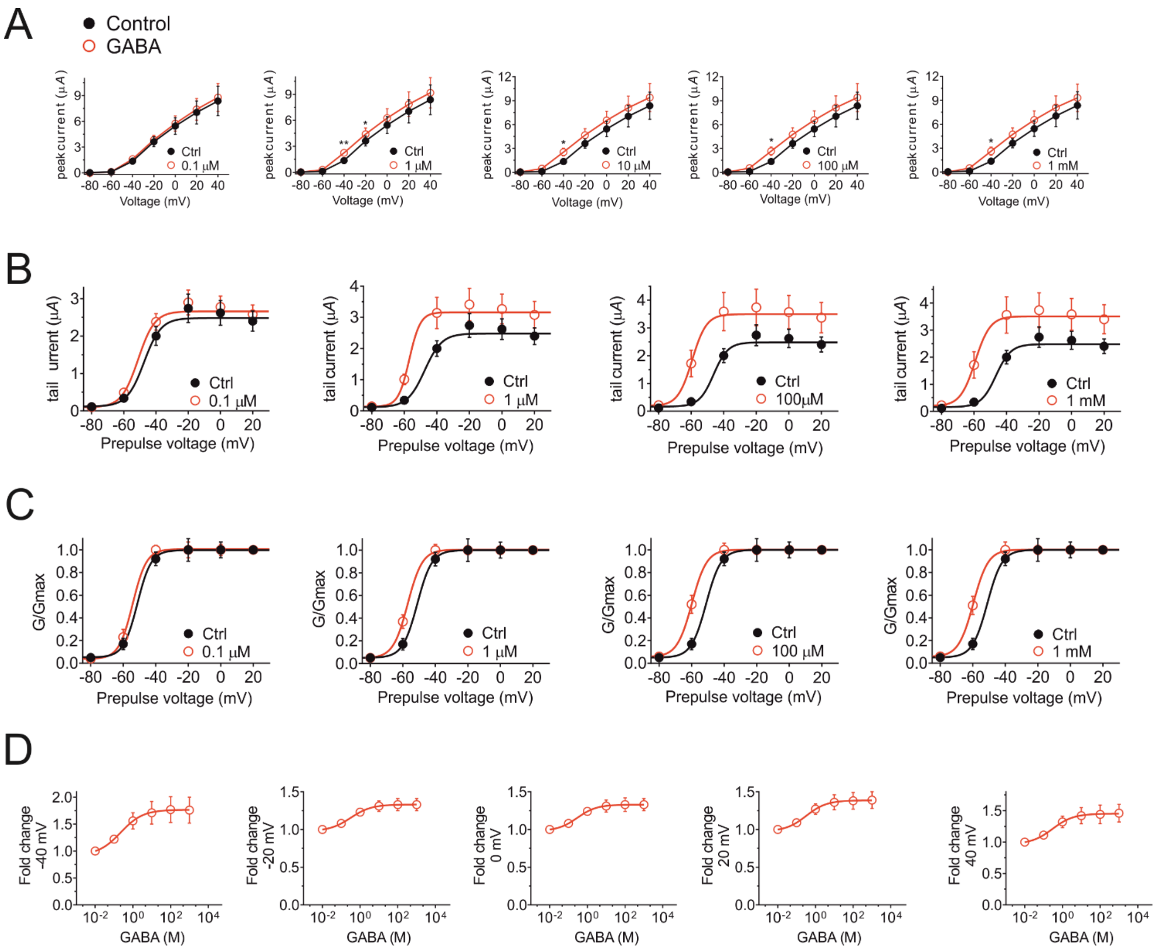

Supplementary Figure 4. Effects of GABA on KCNQ3*

A. Mean peak current-voltage relationship for KCNQ3* channels in the absence (black) and presence (purple) of GABA, *n* = 5, **P=0.005, *P=0.05 versus control. Error bars indicate SEM.

B. Mean tail current versus prepulse voltage relationships as in panel A, *n* = 5. Error bars indicate SEM.

C. Mean normalized tail current versus prepulse voltage relationships as in panel A, *n* = 5. Error bars indicate SEM.

D. Mean dose response of KCNQ3* channels between -40 and +40 mV, *n* = 5. Error bars indicate SEM.

**Q3***	**Normalized tail current $V_{0.5}$ (mV)**	**Non-normalized tail current $V_{0.5}$ (mV)**	**Slope (mV)**
**Ctrl**	-51.4 ± 1.9 (n =5)	-47.5 ± 4.4 (n =5)	4.5 ± 0.9 (n =5)
**0.1 µM GABA**	-54.2 ± 1.5 (n =5)	-51.1 ± 3.5 (n =5)	4.6 ± 0.9 (n =5)
**1 µM GABA**	-56.9 ± 1.0 (n =5) *	-56.9 ± 2.8 (n =5)	4.3 ± 0.8 (n =5)
**10 µM GABA**	-58.9 ± 1.0 (n =5) **	-58.4 ± 2.7 (n =5)	4.3 ± 3.5 (n =5)
**100 µM GABA**	-59.8 ± 0.9 (n =5) **	-59.4 ± 2.8 (n =5)	4.8 ± 3.0 (n =5)
**1 mM GABA**	-59.6 ± 0.9 (n =5) **	-59.3 ± 2.5 (n =5)	4.7 ± 3.3 (n =5)

Supplementary Table 3. Summary of Effects of GABA on KCNQ3* channels.

Statistics versus same channel in absence of GABA: **p=0.004, *p=0.03. Values indicate mean ± SEM.

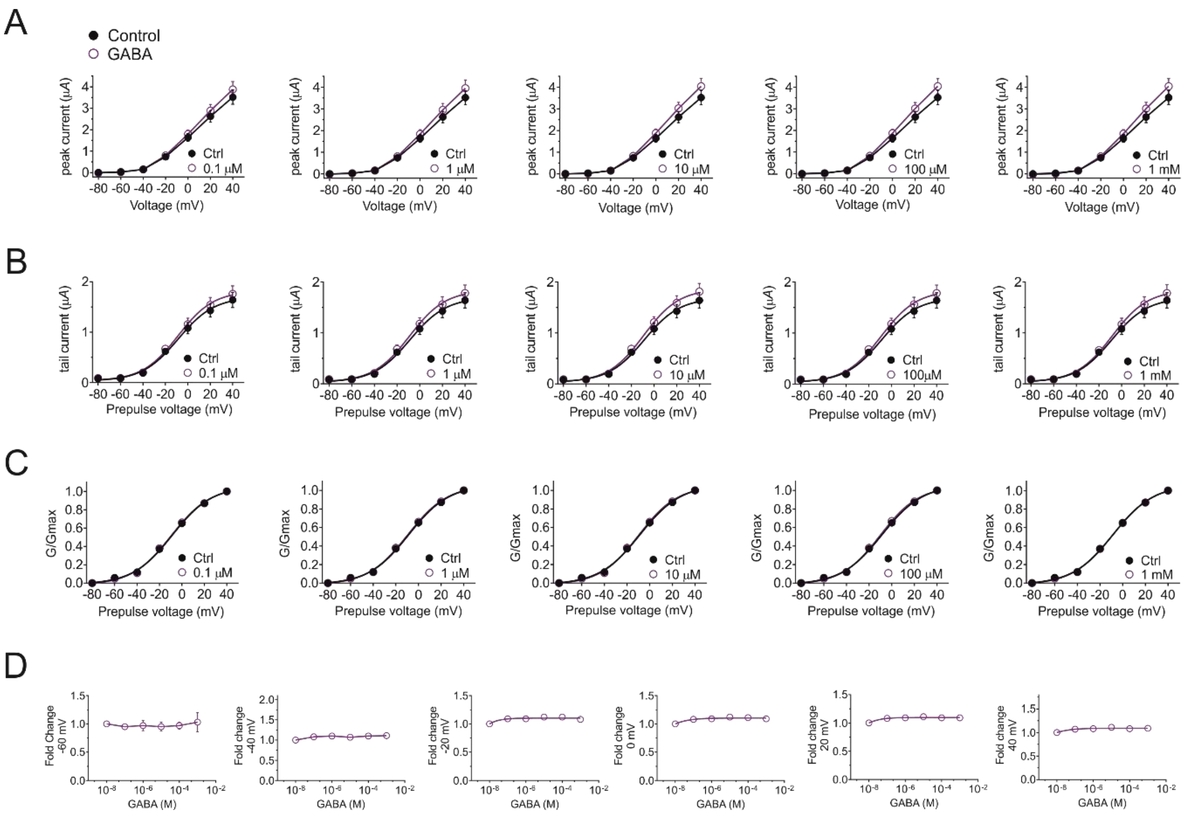

Supplementary Figure 5. Effects of GABA on KCNQ4

A. Mean peak current-voltage relationship for KCNQ4 channels in the absence (black) and presence (purple) of GABA, *n* = 5. Error bars indicate SEM.
B. Mean tail current versus prepulse voltage relationships as in panel A, *n* = 5. Error bars indicate SEM.
C. Mean normalized tail current versus prepulse voltage relationships as in panel A, *n* = 5. Error bars indicate SEM.
D. Mean dose response of KCNQ4 channels between -40 and +40 mV, *n* = 5. Error bars indicate SEM.

**Q4**	**Normalized tail current $V_{0.5}$ (mV)**	**Non-normalized tail current $V_{0.5}$ (mV)**	**Slope (mV)**
**Ctrl**	-8.7 ± 0.8 (n =5)	8.6 ± 4.3 (n =5)	17.6 ± 0.5 (n =5)
**0.1 µM GABA**	-9.6 ± 0.7 (n =5)	-8.8 ± 4.3 (n =5)	17.6 ± 0.5 (n =5)
**1 µM GABA**	-9.6 ± 0.8 (n =5)	-9.0 ± 4.3 (n =5)	17.7 ± 0.6 (n =5)
**10 µM GABA**	-9.8 ± 0.8 (n =5)	-9.3 ± 3.7 (n =5)	17.1 ± 0.8 (n =5)
**100 µM GABA**	-10.3 ± 0.8 (n =5)	-9.0 ± 3.8 (n =5)	17.1 ± 0.9 (n =5)
**1 mM GABA**	-8.8 ± 0.8 (n =5)	-8.4 ± 3.4 (n =5)	17.2 ± 0.8 (n =5)

Supplementary Table 4. Summary of Effects of GABA on KCNQ4 channels.

Values indicate mean ± SEM.

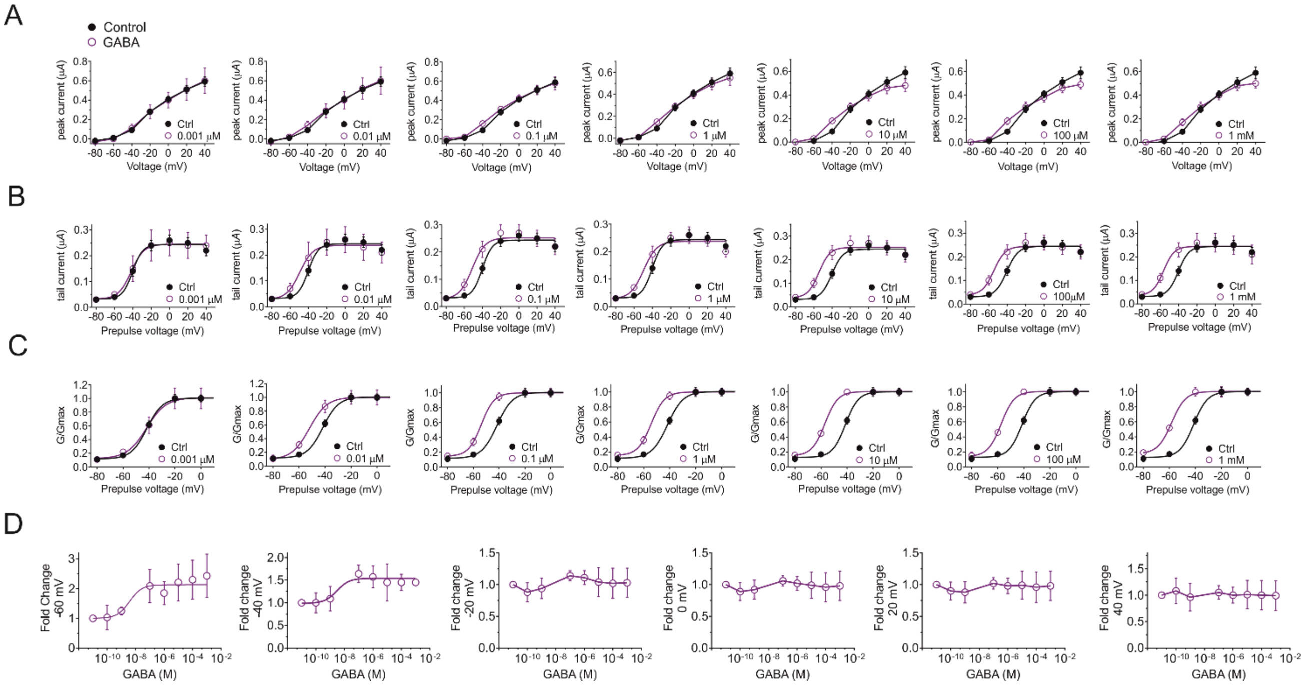

Supplementary Figure 6. Effects of GABA on KCNQ5

A. Mean peak current-voltage relationship for KCNQ5 channels in the absence (black) and presence (purple) of GABA, *n* = 4-8. Error bars indicate SEM.
B. Mean tail current versus prepulse voltage relationships as in panel A, *n* = 4-8. Error bars indicate SEM.
C. Mean normalized tail current versus prepulse voltage relationships as in panel A, *n* = 4-8. Error bars indicate SEM.
D. Mean dose response of KCNQ5 channels between -40 and +40 mV, *n* = 4-8. Error bars indicate SEM.

**Q5**	**Normalized tail current $V_{0.5}$ (mV)**	**Non-normalized tail current $V_{0.5}$ (mV)**	**Slope (mV)**
**Ctrl**	-41.7 ± 1.5 (n =8)	-40.3 ± 2.1 (n =8)	9.6 ± 1.9 (n =8)
**0.001 µM GABA**	-41.9 ± 2.9 (n =4)	-41.8 ± 2.8 (n =4)	9.4 ± 4.3 (n =4)
**0.01 µM GABA**	-50.1 ± 2.9 (n =4) *	-49.5 ± 2.5 (n =4) *	7.6 ± 2.4 (n =4)
**0.1 µM GABA**	-53.9 ± 1.7 (n =8) **	-51.3 ± 2.1 (n =8) *	7.4 ± 1.2 (n =8)
**1 µM GABA**	-54.2 ± 1.6 (n =8) **	-51.2 ± 2.6 (n =8) *	7.6 ± 1.2 (n =8)
**10 µM GABA**	-57.0 ± 1.8 (n =4) **	-54.5 ± 2.3 (n =4) **	8.4 ± 1.4 (n =4)
**100 µM GABA**	-57.4 ± 1.8 (n =4) **	-54.5 ± 2.7 (n =4) **	8.5 ± 1.5 (n =4)
**1 mM GABA**	-58.3 ± 1.8 (n =4) **	-56.4 ± 2.4 (n =4) **	8.8 ± 1.5 (n =4)

Supplementary Table 5. Summary of Effects of GABA on KCNQ5 channels.

Statistics versus same channel in absence of GABA: **p=0.003, *p=0.04. Values indicate mean ± SEM.

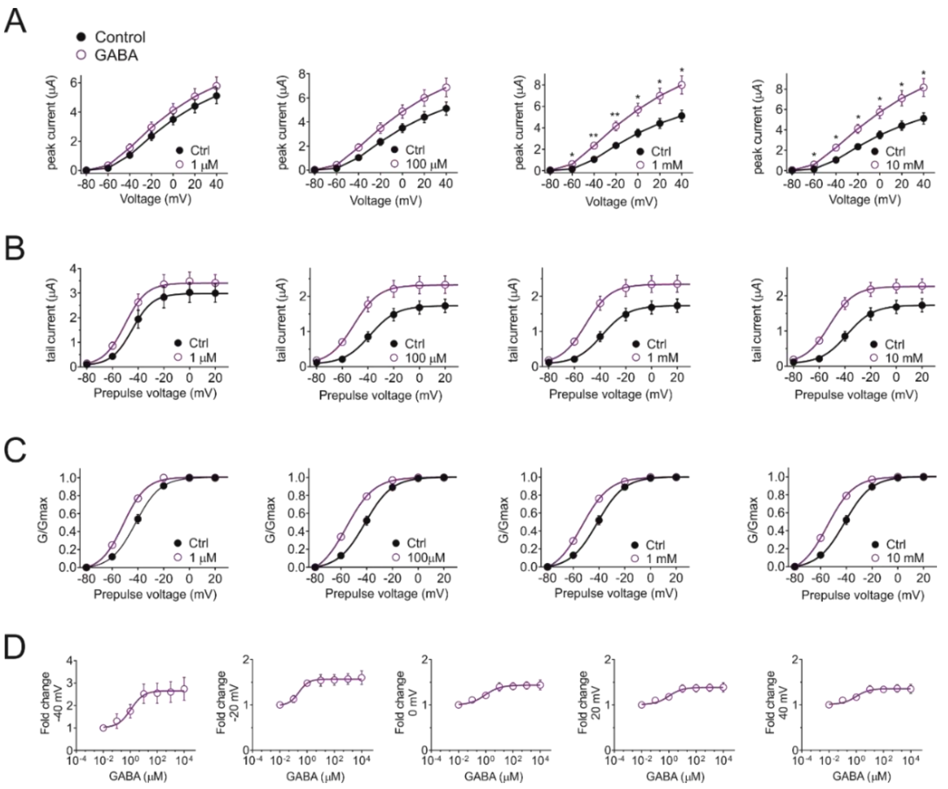

Supplementary Figure 7. Effects of GABA on KCNQ2/3 channels

A. Mean peak current-voltage relationship for KCNQ2/3 channels in the absence (black) and presence (purple) of GABA, *n* = 10, **P=0.007, *P=0.01 versus control. Error bars indicate SEM.

B. Mean tail current versus prepulse voltage relationships as in panel A, *n* = 10. Error bars indicate SEM.

C. Mean normalized tail current versus prepulse voltage relationships as in panel A, *n* = 10. Error bars indicate SEM.

D. Mean dose response of KCNQ3* channels between -40 and +40 mV, *n* = 10. Error bars indicate SEM.

**Q2/Q3**	**Normalized tail current $V_{0.5}$ (mV)**	**Non-normalized tail current $V_{0.5}$ (mV)**	**Slope (mV)**
**Ctrl**	-39.1 ± 1.1 (n =10)	-38.2 ± 4.4 (n =10)	8.4 ± 1.0 (n =10)
**0.1 µM GABA**	-39.8 ± 1.3 (n =4)	-38.4 ± 4.6 (n =4)	8.3 ± 0.9 (n =4)
**1 µM GABA**	-51.3 ± 1.1 (n =10) ****	-51.2 ± 6.1 (n =10)	8.8 ± 0.8 (n =4)
**10 µM GABA**	-53.0 ± 1.1 (n =10) ****	-51.0 ± 5.2 (n =10)	10.9 ± 0.8 (n =10)
**100 µM GABA**	-53.0 ± 0.8 (n =10) ****	-51.0 ± 4.9 (n =10)	10.9 ± 0.6 (n =10)
**1 mM GABA**	-53.1 ± 0.7 (n =10) ****	-51.2 ± 4.2 (n =10) *	10.6 ± 0.6 (n =10)
**10 mM GABA**	-55 ± 0.7 (n =10) ****	-50.3 ± 4.3 (n =10) *	10.3 ± 0.6 (n =10)

Supplementary Table 6. Summary of Effects of GABA on KCNQ2/3 channels.

Statistics versus same channel in absence of GABA:

****p<0.0001, *p=0.02. Values indicate mean ± SEM.

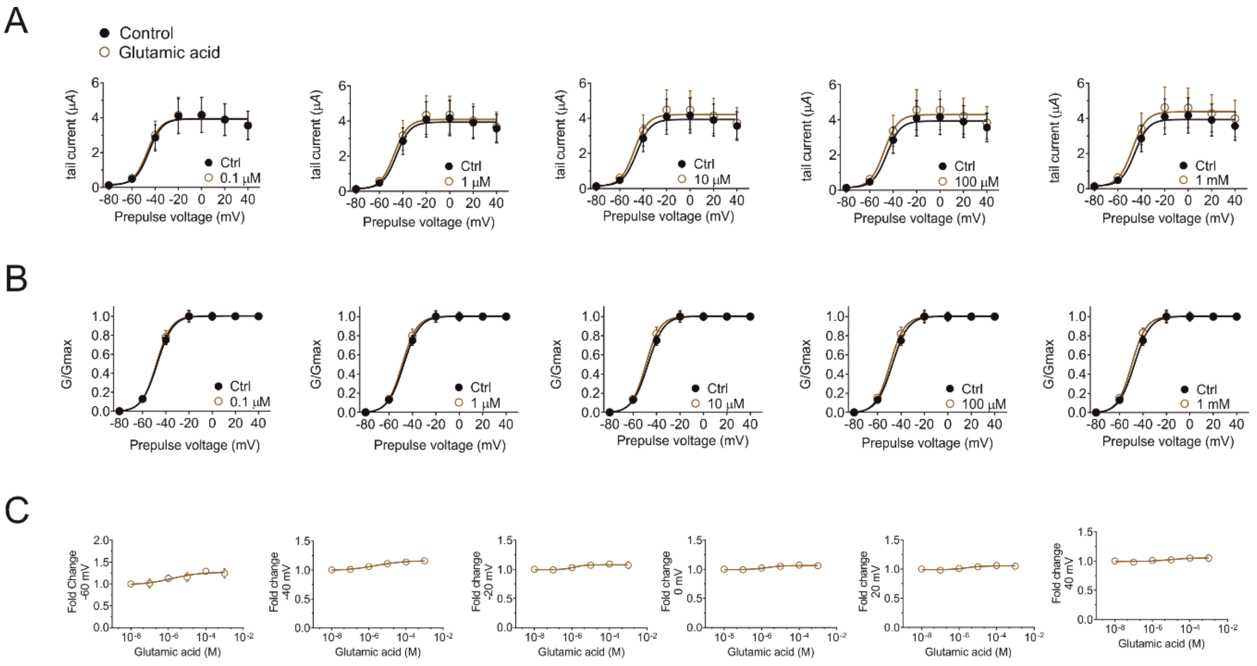

Supplementary Figure 8. Glutamate has no effect on KCNQ2/3 channels

A. Mean tail current versus prepulse voltage relationships for KCNQ2/3 channels in the absence (black) and presence (brown) of glutamate, *n* = 5. Error bars indicate SEM.
B. Mean normalized tail current versus prepulse voltage relationships as in panel A, *n* = 5. Error bars indicate SEM.
C. Mean dose response of KCNQ2/3 channels between -40 and +40 mV, *n* = 5. Error bars indicate SEM.

**Q2/Q3**	**Normalized tail current $V_{0.5}$ (mV)**	**Non-normalized tail current $V_{0.5}$ (mV)**	**Slope (mV)**
**Ctrl**	-47.4 ± 1.4 (n =5)	-45.9 ± 8.4 (n =5)	6.7 ± 0.9 (n =5)
**0.1 µM Glutamate**	-48.1 ± 1.6 (n =5)	-46.9 ± 5.7 (n =5)	6.4 ± 1.0 (n =5)
**1 µM Glutamate**	-48.8 ± 1.7 (n =5)	-47.4 ± 5.5 (n =5)	6.3 ± 1.7 (n =5)
**10 µM Glutamate**	-49.2 ± 1.6 (n =5)	-47.6 ± 5.4 (n =5)	6.1 ± 0.9 (n =5)
**100 µM Glutamate**	-49.5 ± 1.8 (n =5)	-47.7 ± 5.3 (n =5)	6.2 ± 1.0 (n =5)
**1 mM Glutamate**	-49.7 ± 1.6 (n =5)	-47.6 ± 5.6 (n =5)	6.1 ± 0.9 (n =5)

Supplementary Table 7. Summary of Effects of glutamate on KCNQ2/3 channels.

Values indicate mean ± SEM.

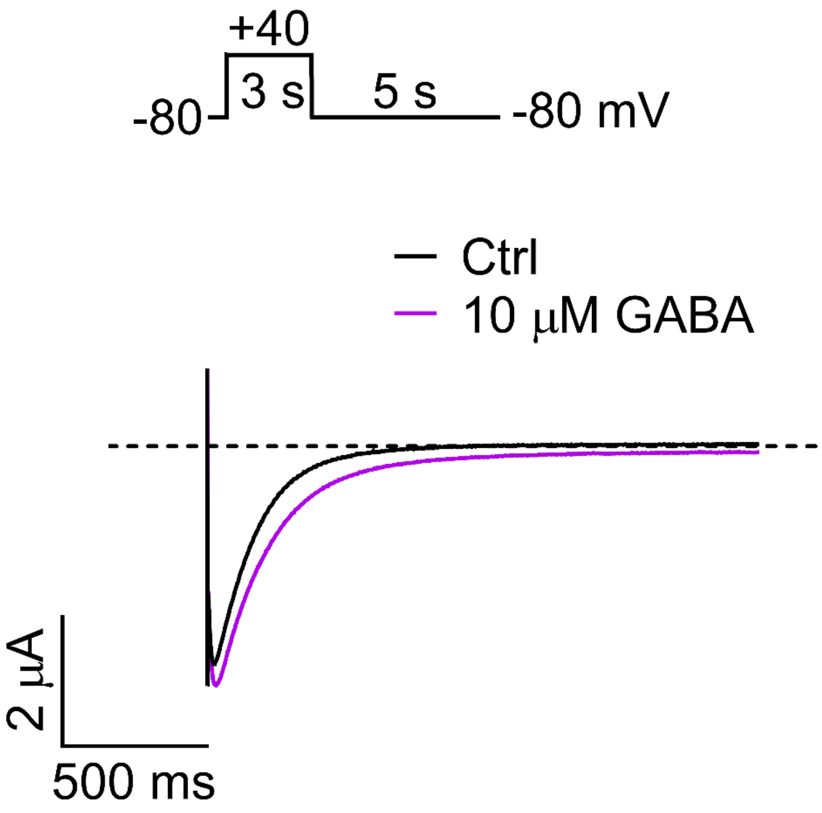

Supplementary Figure 9. Exemplar traces of effects of GABA on KCNQ2/3 deactivation
KCNQ2/3 deactivation at -80 mV after a 40 mV prepulse, using the voltage protocol (upper) in the absence or presence of GABA (10 μM). See Figure 2k and Supplementary Table 7 for mean data.

KCNQ2/3	τ act, -40 mV (ms)	τ deact, -80 mV (ms)
**Ctrl**	1229 ± 153 (n =10)	156 ± 10 (n =10)
**10 μM GABA**	701 ± 64 ** (n =10)	219 ± 11 *** (n =10)

Supplementary Table 8. Mean effects of GABA on KCNQ2/3 gating kinetics.

Statistics versus same channel in absence of GABA:

**p<0.01; ***p<0.001. Values indicate mean ± SEM.

GABA	EC_{50} (µM)
**Q2/Q3**	0.85 ± 0.1<br>(n = 10)
**Q2/Q3<br>Wort**	1.2 ± 0.12<br>(n = 5)
**Q3***	1.0 ± 0.1<br>(n = 5)
**Q5**	0.06 ± 0.01<br>(n = 4-8)

BHB	EC_{50} (µM)
**Q2/Q3**	0.95 ± 0.13<br>(n = 5)

GABOB	EC_{50} (µM)
**Q2/Q3**	0.1 ± 0.01<br>(n = 6)

Supplementary Table 9. EC_{50} values calculated from dose responses at -60 mV. Values indicate mean ± SEM.

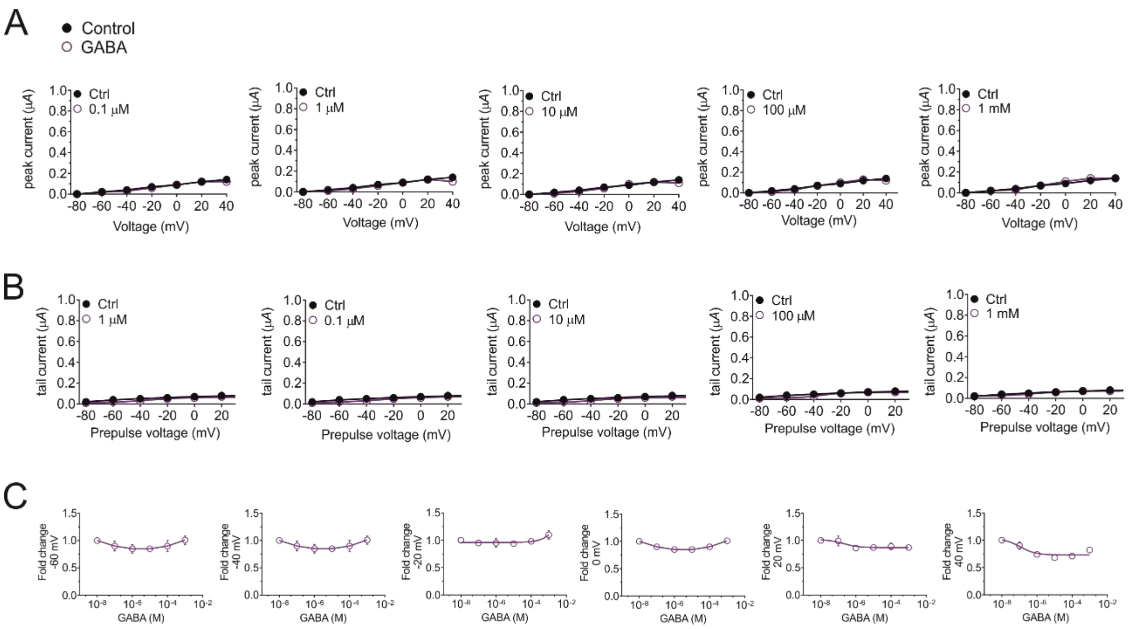

Supplementary Figure 10. Lack of effects of GABA on H_2O-injected oocytes

A. Mean peak current-voltage relationship for H_20 injected oocytes in the absence (black) and presence (purple) of GABA, *n* = 4. Error bars indicate SEM.

B. Mean normalized tail current versus prepulse voltage relationships as in panel A, *n* = 4. Error bars indicate SEM.

C. Mean dose response of H_20 injected oocytes between -60 and +40 mV, *n* = 4. Error bars indicate SEM.

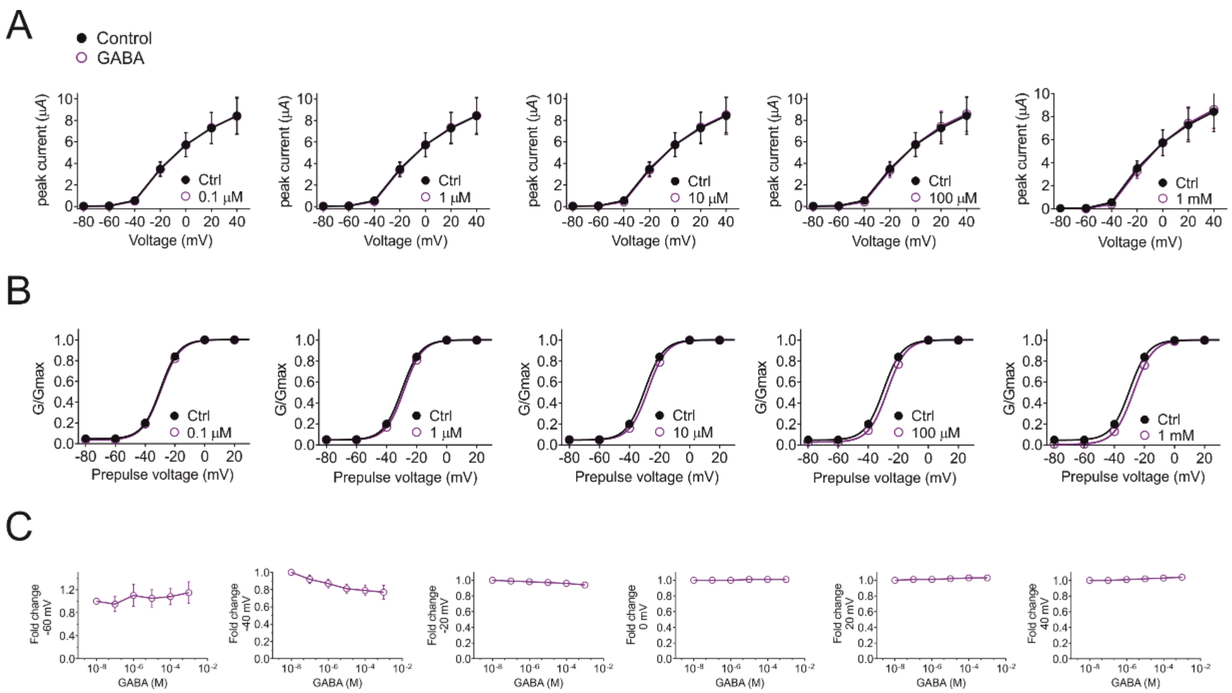

Supplementary Figure 11. Lack of effects of GABA on KCNA1

A. Mean peak current-voltage relationship for KCNA1 channels in the absence (black) and presence (purple) of GABA, *n* = 4. Error bars indicate SEM.

B. Mean normalized tail current versus prepulse voltage relationships as in panel A, *n* = 4. Error bars indicate SEM.

C. Mean dose response of KCNA1 channels between -60 and +40 mV, *n* = 4. Error bars indicate SEM.

**KCNA1**	**Normalized tail current $V_{0.5}$ (mV)**	**Non-normalized tail current $V_{0.5}$ (mV)**	**Slope (mV)**
**Ctrl**	-29.8 ± 0.69 (n =4)	n/a	6.2 ± 0.4 (n =4)
**0.1 µM GABA**	-29.3 ± 0.88 (n =4)	n/a	6.4 ± 0.5 (n =4)
**1 µM GABA**	-28.4 ± 0.89 (n =4)	n/a	6.1 ± 0.5 (n =4)
**10 µM GABA**	-27.6 ± 0.82 (n =4)	n/a	6.1 ± 0.5 (n =4)
**100 µM GABA**	-27.2 ± 0.67 (n =4)	n/a	6.2 ± 0.4 (n =4)
**1 mM GABA**	-27.3 ± 0.93 (n =4)	n/a	6.4 ± 0.6 (n =4)

Supplementary Table 10. Summary of effects of GABA on KCNA1 channels.

Values indicate mean ± SEM.

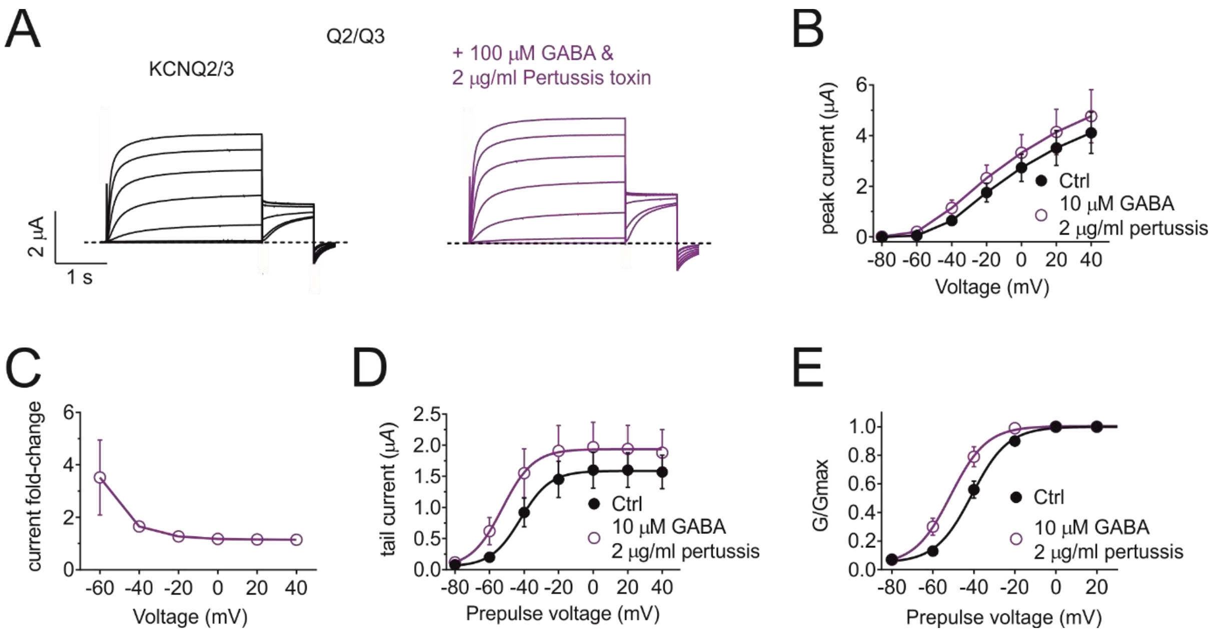

Supplementary Figure 12. Pertussis toxin has no effect on GABA sensitivity of KCNQ2/3 channels

A. Averaged KCNQ2/3 traces in the absence (black) and presence (purple) of GABA (10 μM) and pertussis toxin (2 μg/ml), *n* = 7.
B. Mean peak current-voltage relationship for recordings as in panel A, *n* = 7. Error bars indicate SEM.
C. Mean current fold-change versus voltage as in panel A, *n* = 7. Error bars indicate SEM.
D. Mean tail current versus prepulse voltage relationships as in panel A, *n* = 7. Error bars indicate SEM.
E. Mean normalized tail current versus prepulse voltage relationships as in panel A, *n* = 7. Error bars indicate SEM.

**Q2/Q3**	**Normalized tail current $V_{0.5}$ (mV)**	**Non-normalized tail current $V_{0.5}$ (mV)**	**Slope (mV)**
**Ctrl**<br>**2 μg/ml Pertussis toxin**	-40.9 ± 1.4<br>(n =7)	-41.9 ± 6.3<br>(n =7)	8.8 ± 1.4<br>(n =7)
**100 μM GABA**<br>**2 μg/ml Pertussis toxin**	-51.3 ± 1.9<br>(n =7) **	-52.6 ± 9.1<br>(n =7)	8.8 ± 1.4<br>(n =7)

Supplementary Table 11. Summary of Effects of pertussis toxin on GABA sensitivity of KCNQ2/3 channels.Statistics versus same channel in absence of 100 μM GABA: **p=0.001. Values indicate mean ± SEM.

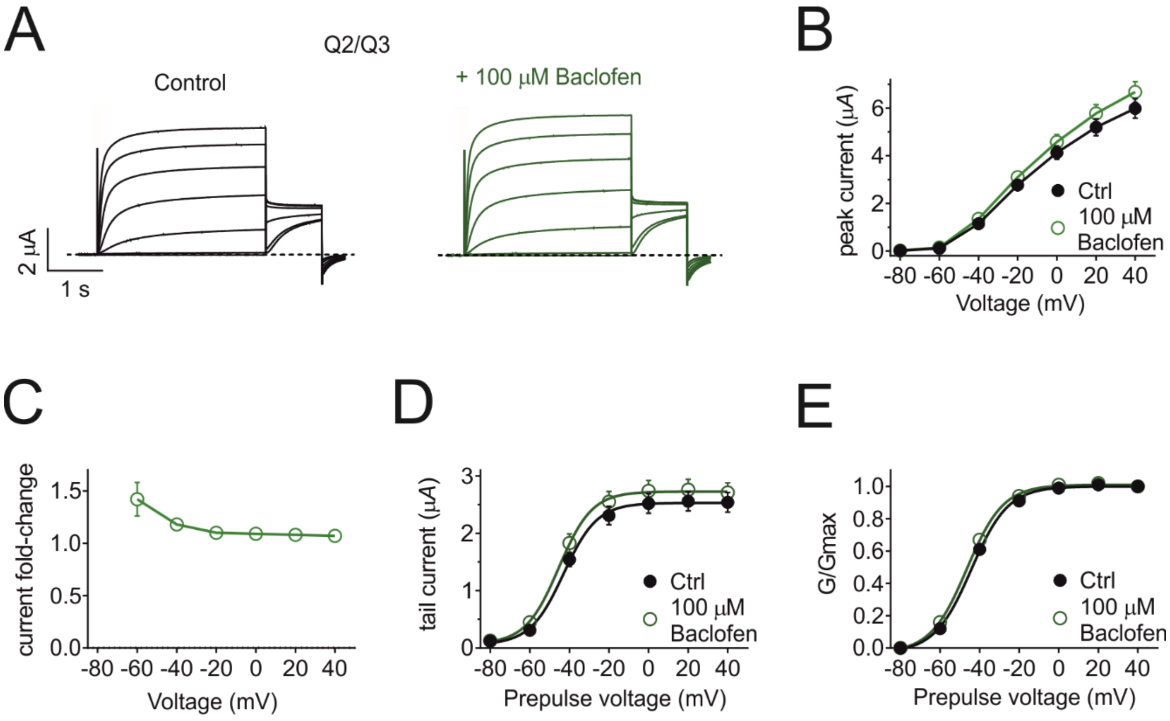

Supplementary Figure 13. Minimal effects of baclofen on KCNQ2/3 channels

A. Averaged KCNQ2/3 traces in the absence (black) and presence (green) of baclofen (100 μM), *n* = 5.

B. Mean peak current-voltage relationship as in panel A, *n* = 5. Error bars indicate SEM.

C. Mean current fold-change versus voltage as in panel A, *n* = 5. Error bars indicate SEM.

D. Mean tail current versus prepulse voltage relationships as in panel A, *n* = 5. Error bars indicate SEM.

E. Mean normalized tail current versus prepulse voltage relationships as in panel A, *n* = 5. Error bars indicate SEM.

**Q2/Q3**	**Normalized tail current $V_{0.5}$ (mV)**	**Non-normalized tail current $V_{0.5}$ (mV)**	**Slope (mV)**
**Ctrl**	-44.2 ± 0.4 (n =5)	-42.6 ± 2.3 (n =5)	9.3 ± 0.3 (n =5)
**100 μM Baclofen**	-46.4 ± 0.6 (n =5)	-45.4 ± 2.5 (n =5)	9.2 ± 0.5 (n =5)

Supplementary Table 12. Summary of Effects of baclofen on KCNQ2/3 channels.

Values indicate mean ± SEM.

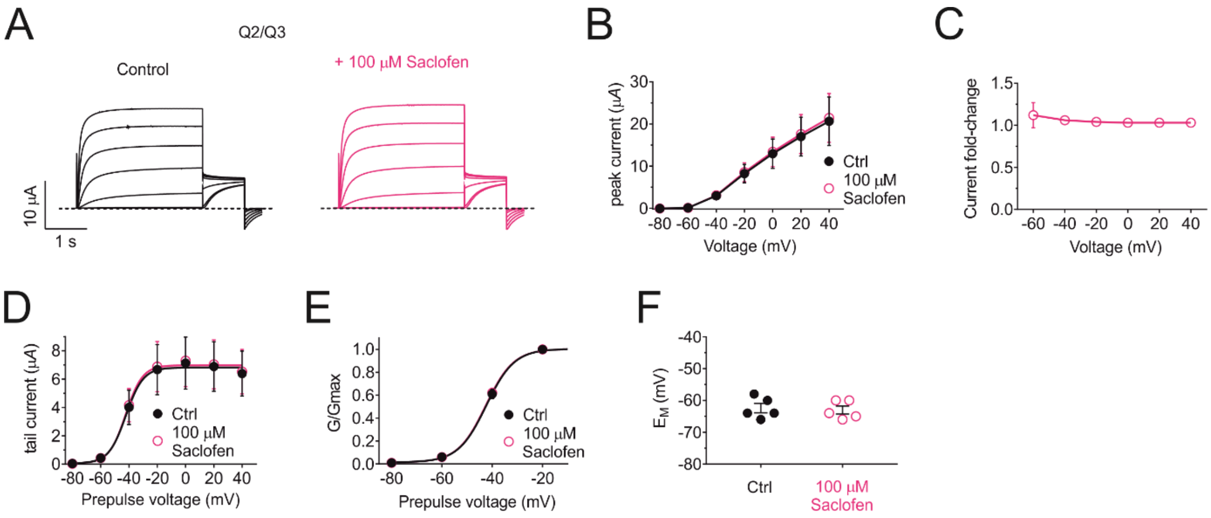

Supplementary Figure 14. Lack of effects of saclofen on KCNQ2/3 channels

A. Averaged KCNQ2/3 traces in the absence (black) and presence (pink) of saclofen (100 µM), *n* = 5.
B. Mean peak current-voltage relationship as in panel A, *n* = 5. Error bars indicate SEM.
C. Mean current fold-change versus voltage as in panel A, *n* = 5. Error bars indicate SEM.
D. Mean tail current versus prepulse voltage relationships as in panel A, *n* = 5. Error bars indicate SEM.
E. Mean normalized tail current versus prepulse voltage relationships as in panel A, *n* = 5. Error bars indicate SEM.
F. Scatter plot of unclamped resting membrane potential (E_M) as in panel A, *n* = 5. Error bars indicate SEM.

Q2/Q3	Normalized tail current $V_{0.5}$ (mV)	Non-normalized tail current $V_{0.5}$ (mV)	Slope (mV)
**Ctrl**	-42.3 ± 0.8 (n =5)	-41.1 ± 6.7 (n =5)	5.6 ± 1.1 (n =5)
**100 µM Saclofen**	-42.6 ± 0.8 (n =5)	-42.8 ± 6.6 (n =5)	5.5 ± 0.8 (n =5)

Supplementary Table 13. Summary of Effects of saclofen on KCNQ2/3 channels.

Values indicate mean ± SEM.

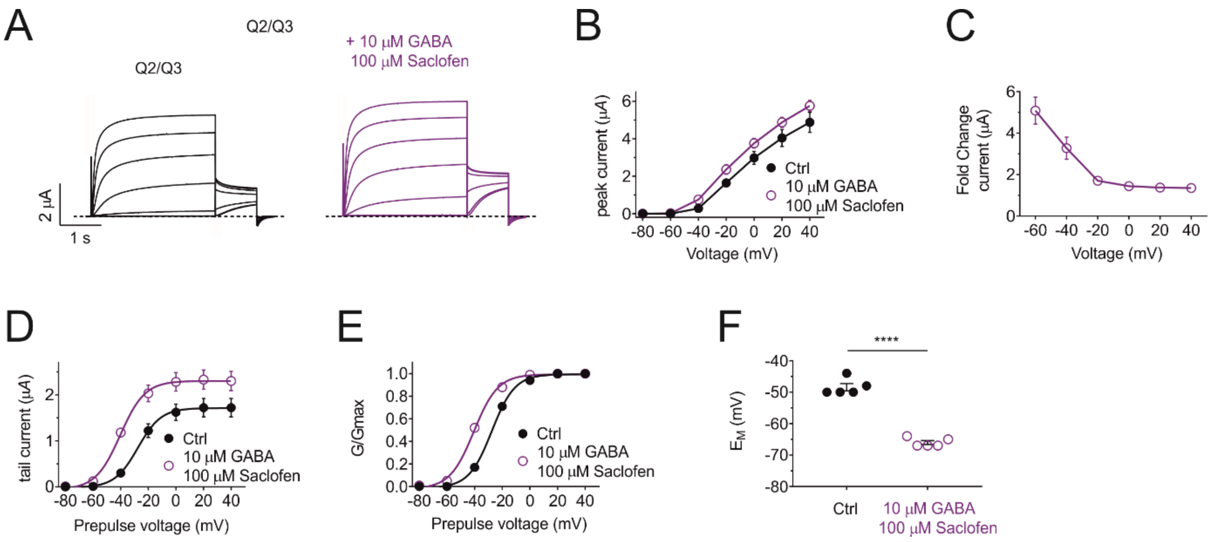

Supplementary Figure 15. Saclofen exhibits no effect on GABA sensitivity of KCNQ2/3 channels

A. Averaged KCNQ2/3 traces in the absence (black) and presence (purple) of GABA (10 µM) and saclofen (100 µM), *n* = 6.
B. Mean peak current-voltage relationship for recordings as in panel A, *n* = 6. Error bars indicate SEM.
C. Mean current fold-change versus voltage as in panel a, *n* = 6. Error bars indicate SEM.
D. Mean tail current versus prepulse voltage relationships as in panel A, *n* = 6. Error bars indicate SEM.
E. Mean normalized tail current versus prepulse voltage relationships as in panel A, *n* = 6. Error bars indicate SEM.
F. Scatter plot of unclamped resting membrane potential (E_M) for oocytes as in panel A, *n* = 6, ****P<0.0001 versus control. Error bars indicate SEM.

Q2/Q3	Normalized tail current $V_{0.5}$ (mV)	Non-normalized tail current $V_{0.5}$ (mV)	Slope (mV)
**Ctrl**	-27.5 ± 0.4 (n =6)	-27.5 ± 3.2 (n =6)	8.3 ± 0.4 (n =6)
**10 µM GABA 100 µM Saclofen**	-40.5 ± 0.5 (n =6) ****	-40.3 ± 2.7 (n =6) *	8.7 ± 0.6 (n =6)

Supplementary Table 14. Summary of Effects of saclofen on GABA sensitivity of KCNQ2/3 channels.

Statistics versus same channel in absence of 10 µM GABA and 100 µM saclofen: ****p<0.0001, *p=0.01.

Values indicate mean ± SEM.

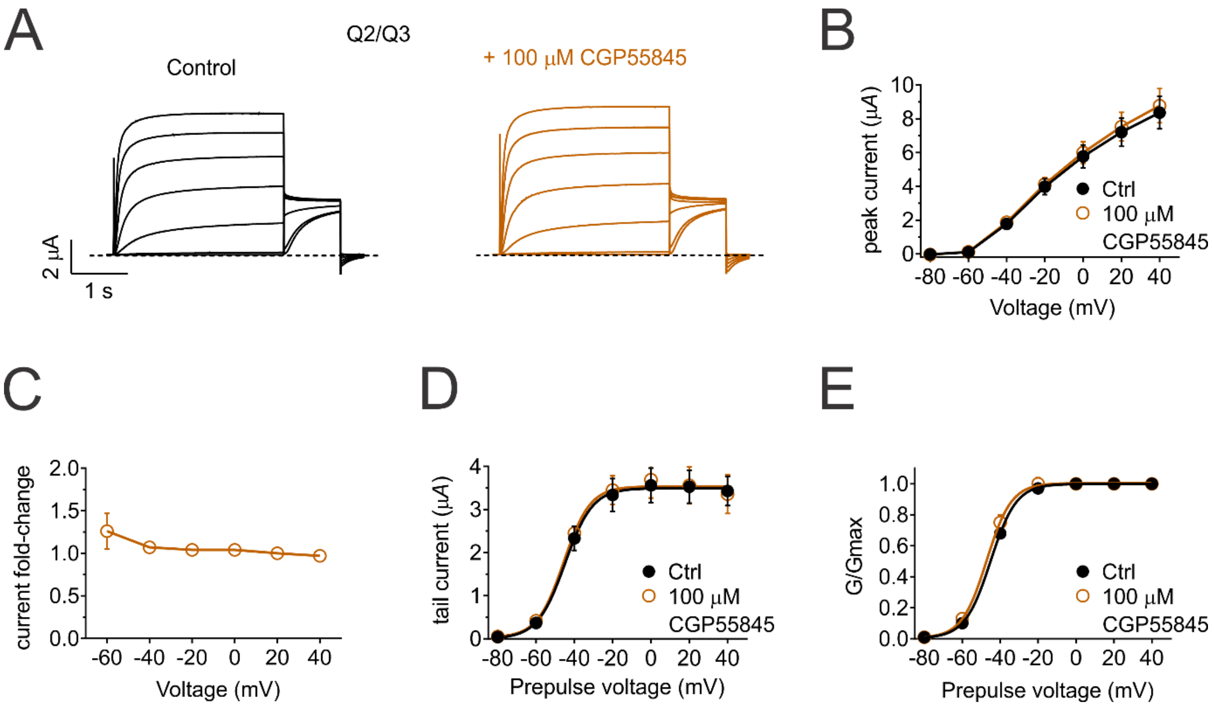

Supplementary Figure 16. Lack of effects of CGP55845 on KCNQ2/3 channels

A. Averaged KCNQ2/3 traces in the absence (black) and presence (brown) of CGP55845 (100 μM), n = 4.
B. Mean peak current-voltage relationship as in panel A, n = 4. Error bars indicate SEM.
C. Mean current fold-change versus voltage as in panel A, n = 4. Error bars indicate SEM.
D. Mean tail current versus prepulse voltage relationships as in panel A, n = 4. Error bars indicate SEM.
E. Mean normalized tail current versus prepulse voltage relationships as in panel A, n = 4. Error bars indicate SEM.

**Q2/Q3**	**Normalized tail current $V_{0.5}$ (mV)**	**Non-normalized tail current $V_{0.5}$ (mV)**	**Slope (mV)**
**Ctrl**	-45.1 ± 0.3 (n =4)	-44.9 ± 3.5 (n =4)	6.7 ± 0.3 (n =4)
**100 μM CGP**	-47.1 ± 1.1 (n =4)	-45.6 ± 3.7 (n =4)	6.6 ± 0.7 (n =4)

Supplementary Table 15. Summary of Effects of CGP55485 on KCNQ2/3 channels.

Values indicate mean ± SEM.

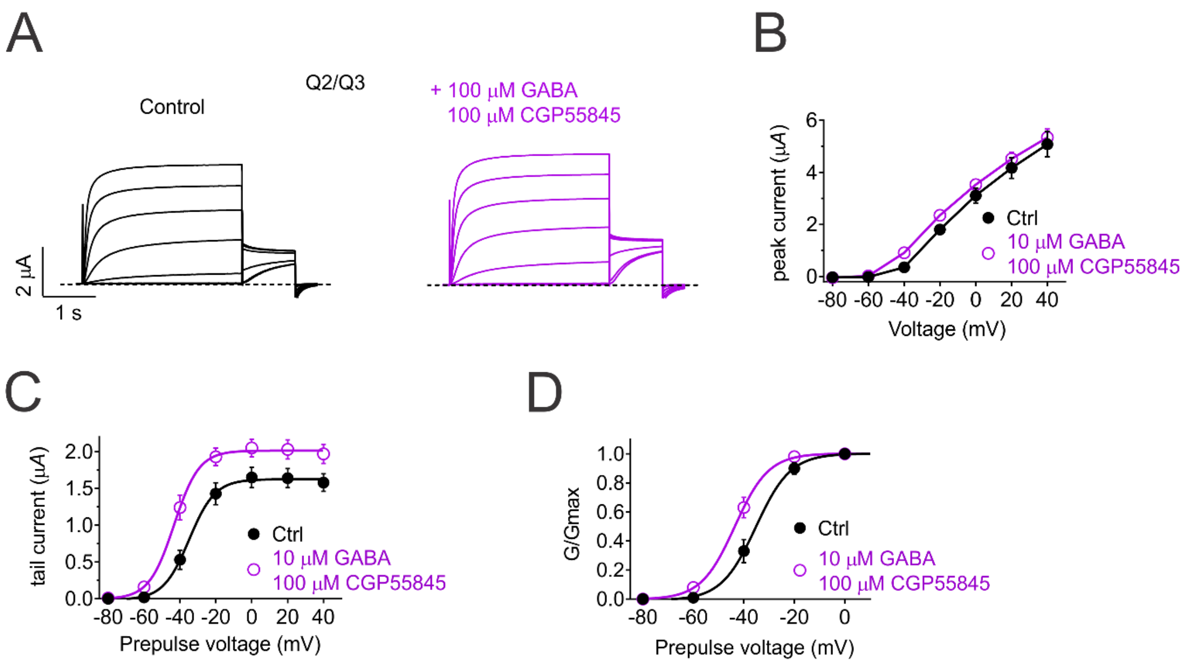

Supplementary Figure 17. CGP55845 exhibits no effect on GABA sensitivity of KCNQ2/3 channels

A. Averaged KCNQ2/3 traces in the absence (black) and presence (purple) of GABA (10 μM) and CGP55845 (100 μM), *n* = 5.
B. Mean peak current-voltage relationship for recordings as in panel A, *n* = 5. Error bars indicate SEM.
C. Mean tail current versus prepulse voltage relationships as in panel A, *n* = 5. Error bars indicate SEM.
D. Mean normalized tail current versus prepulse voltage relationships as in panel A, *n* = 5. Error bars indicate SEM.

Q2/Q3	Normalized tail current $V_{0.5}$ (mV)	Non-normalized tail current $V_{0.5}$ (mV)	Slope (mV)
**Ctrl**	-35.3 ± 1.2 (n =5)	-34.9 ± 25 (n =5)	6.9 ± 1.1 (n =5)
**10 μM GABA 100 μM CGP**	-43.6 ± 1.1 (n =5) ***	-43.3 ± 2.1 (n =5) *	6.6 ± 1.1 (n =5)

Supplementary Table 16. Summary of Effects of CGP55485 on GABA sensitivity of KCNQ2/3 channels.

Statistics versus same channel in absence of 10 μM GABA and 100 μM CGP55485: ***p=0.001, *p=0.03. Values indicate mean ± SEM.

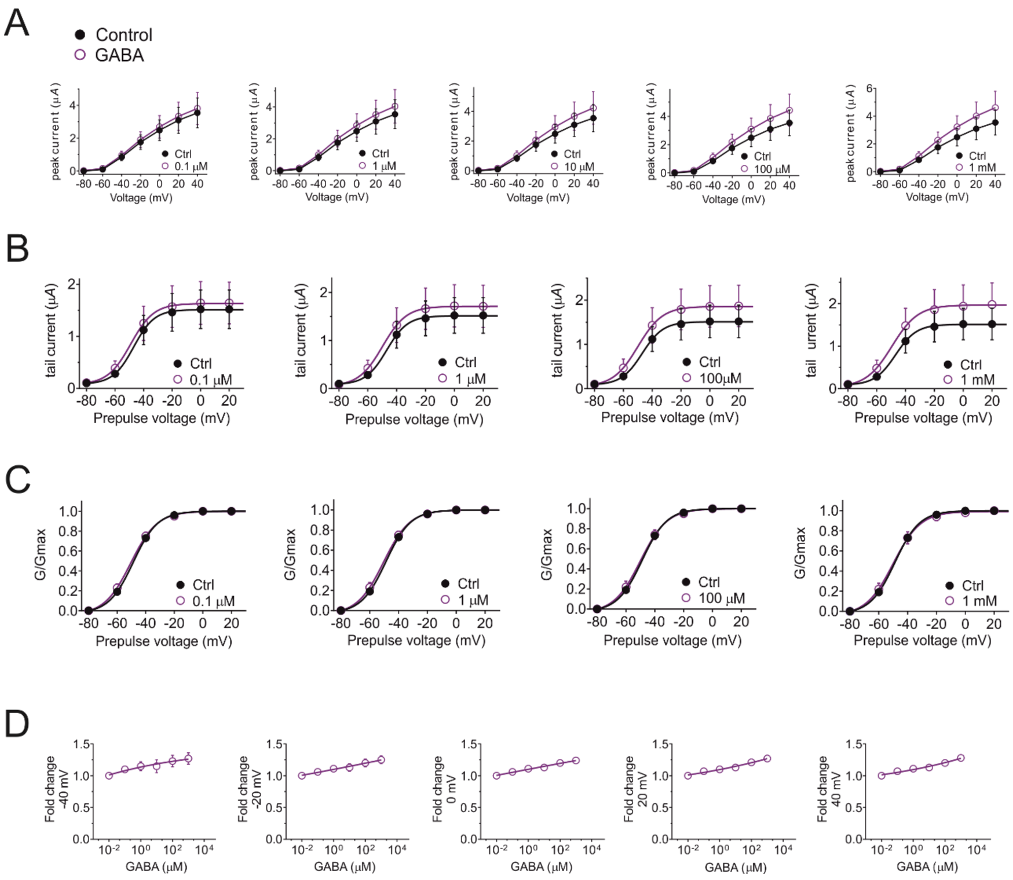

Supplementary Figure 18. Q2/Q3-W265L has diminished sensitivity to GABA

A. Mean peak current-voltage relationship for W265L-Q3/Q2 channels in the absence (black) and presence (purple) of GABA, *n* = 5. Error bars indicate SEM.

B. Mean tail current versus prepulse voltage relationships as in panel A, *n* = 5. Error bars indicate SEM.

C Mean normalized tail current versus prepulse voltage relationships as in panel A, *n* = 5. Error bars indicate SEM.

D. Mean dose response of W265L-Q3/Q2 channels between -40 and +40 mV, *n* = 5. Error bars indicate SEM.

**Q2/W265L-Q3**	**Normalized tail current $V_{0.5}$ (mV)**	**Non-normalized tail current $V_{0.5}$ (mV)**	**Slope (mV)**
**Ctrl**	-48.8 ± 0.6 (n =5)	-46.6 ± 8.7 (n =5)	8.6 ± 0.4 (n =5)
**0.1 μM GABA**	-50.5 ± 1.2 (n =5)	-49.0 ± 9.7 (n =5)	9.2 ± 0.9 (n =5)
**1 μM GABA**	-50.4 ± 1.3 (n =5)	-49.7 ± 10 (n =5)	9.1 ± 0.9 (n =5)
**10 μM GABA**	-49.9 ± 1.5 (n =5)	-49.9 ± 10.4 (n =5)	9.1 ± 1.1 (n =5)
**100 μM GABA**	-49.9 ± 1.5 (n =5)	-49.9 ± 10.2 (n =5)	9.2 ± 1.4 (n =5)
**1 mM GABA**	-49.9 ± 1.9 (n =5)	-49.4 ± 10.7 (n =5)	9.4 ± 1.5 (n =5)

Supplementary Table 17. Summary of Effects of GABA on Q2-W265L-Q3 channels.

Values indicate mean ± SEM.

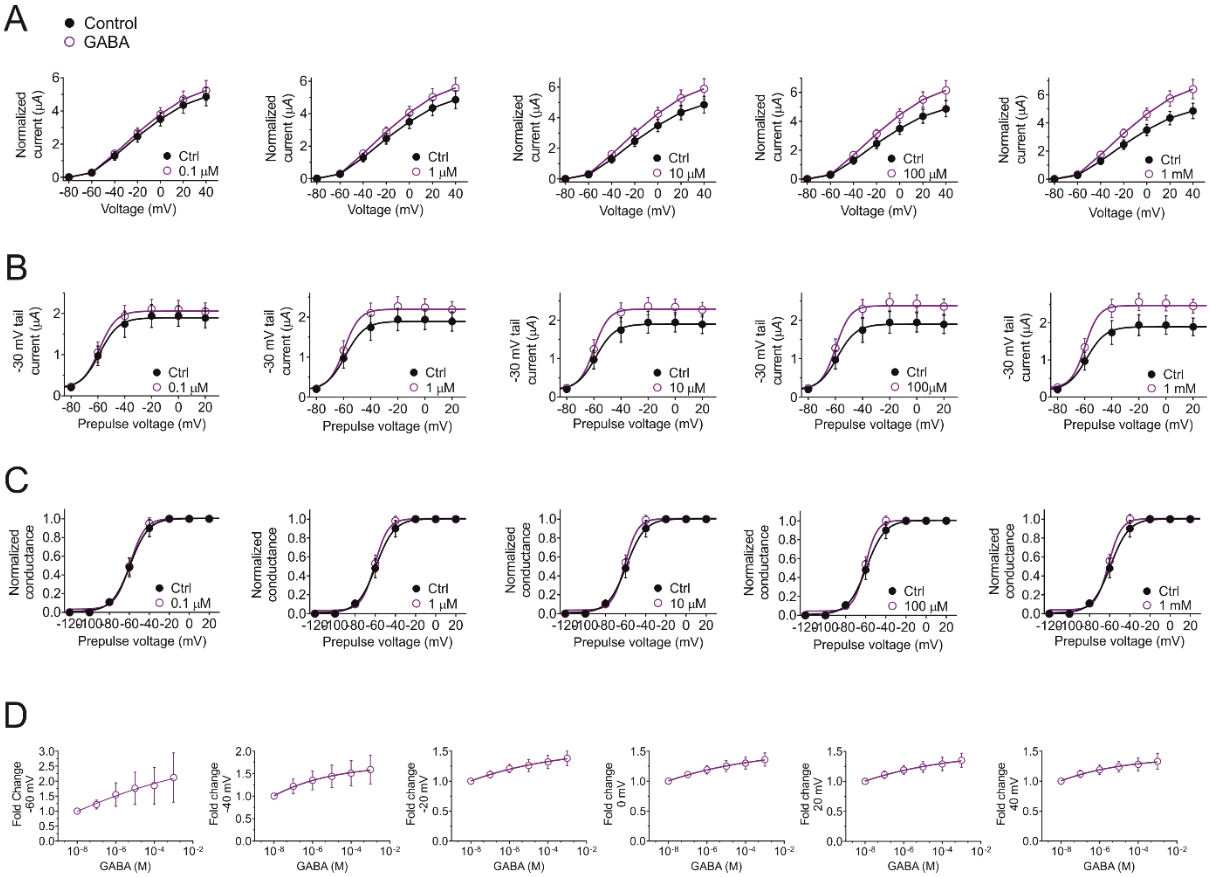

Supplementary Figure 19. W236L-Q2/W265L-Q3 heteromers show reduced GABA sensitivity

A. Mean peak current-voltage relationship for W236L-Q2/W265L-Q3 channels in the absence (black) and presence (purple) of GABA, *n* = 5. Error bars indicate SEM.

B. Mean tail current versus prepulse voltage relationships as in panel A, *n* = 5. Error bars indicate SEM.

C Mean normalized tail current versus prepulse voltage relationships as in panel A, *n* = 5. Error bars indicate SEM.

D. Mean dose response of W236L-Q2/W265L-Q3 channels between -40 and +40 mV, *n* = 5. Error bars indicate SEM.

Q2/Q3	Normalized tail current $V_{0.5}$ (mV)	Slope (mV)
**Ctrl**	-2.9 ± 2.7 (n = 6)	14.3 ± 1.6 (n = 6)
**100 µM GABA**	-17.5 ± 3.7 (n = 6)	16.2 ± 3.0 (n = 6)

Supplementary Table 18. Summary of effects of 100 µM GABA on KCNQ2/3 expressed in CHO cells.

Values indicate mean ± SEM.

Q2/Q3	Normalized tail current $V_{0.5}$ (mV)	Slope (mV)
**Ctrl (extracellular solution only)**	-9.2 ± 4.9	20.3 ± 4.5
**100 µM picrotoxin 10 µM CGP55845**	-7.1 ± 7.6	17.4 ± 3.0
**100 µM picrotoxin 10 µM CGP55845 100 µM GABA**	-20.6 ± 4.9	16.0 ± 3.2
**100 µM picrotoxin 10 µM CGP55845 10 µM XE991**	-4.9 ± 4.3	18.4 ± 2.2

Supplementary Table 19. Summary of effects of 100 µM GABA on native M-current in PC12 cells.

Recordings performed for each condition, in order, on each cell (top to bottom in table); *n* = 7 cells. Values indicate mean ± SEM.

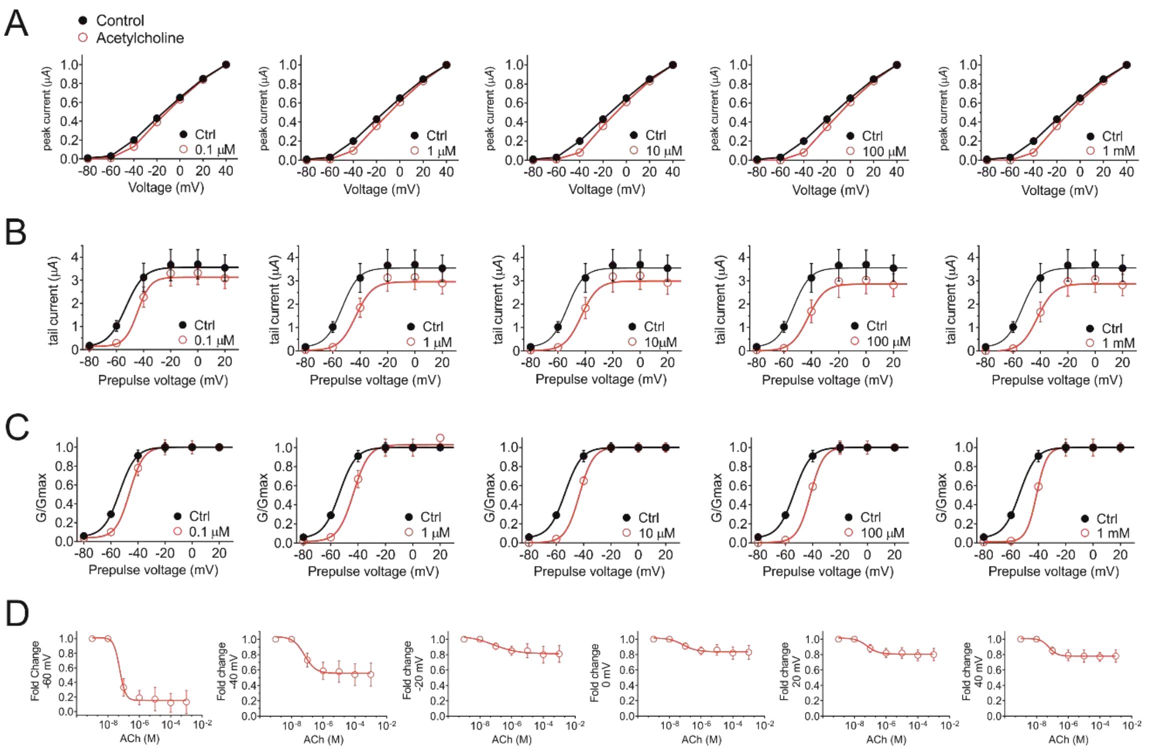

Supplementary Figure 20. Acetylcholine effects on KCNQ2/3 channels

A. Mean normalized peak current-voltage relationship for KCNQ2/Q3 channels in the absence (black) and presence (red) of Acetylcholine, *n* = 5. Error bars indicate SEM.

B. Mean tail current versus prepulse voltage relationships as in panel A, *n* = 5. Error bars indicate SEM.

C Mean normalized tail current versus prepulse voltage relationships as in panel A, *n* = 5. Error bars indicate SEM.

D. Mean dose response of KCNQ2/3 channels between -60 and +40 mV, *n* = 5. Error bars indicate SEM.

Q2/Q3	Normalized tail current $V_{0.5}$ (mV)	Non-normalized tail current $V_{0.5}$ (mV)	Slope (mV)
**Ctrl**	-53.5 ± 1.6 (n =5)	-53.1 ± 5.8 (n =5)	6.0 ± 0.9 (n =5)
**0.1 µM ACh**	-46.2 ± 2.5 (n =5) *	-44.4 ± 6.3 (n =5)	5.1 ± 2.5 (n =5)
**1 µM ACh**	-43.4 ± 2.4 (n =5) *	-43.2 ± 4.7 (n =5)	5.6 ± 2.7 (n =5)
**10 µM ACh**	-43.1 ± 1.5 (n =5) **	-42.9 ± 4.7 (n =5)	5.1 ± 1.9 (n =5)
**100 µM ACh**	-41.7 ± 1.4 (n =5) ***	-42.4 ± 4.8 (n =5)	4.8 ± 2.7 (n =5)
**1 mM ACh**	-41.3 ± 2.5 (n =5) **	-42.2 ± 4.6 (n =5)	3.8 ± 6.5 (n =5)

Supplementary Table 20. Summary of effects of acetylcholine on KCNQ2/3 channels.

Statistics versus same channel in absence of acetylcholine: ***p=0.006, **p=0.001, *p=0.04. Values indicate mean ± SEM.

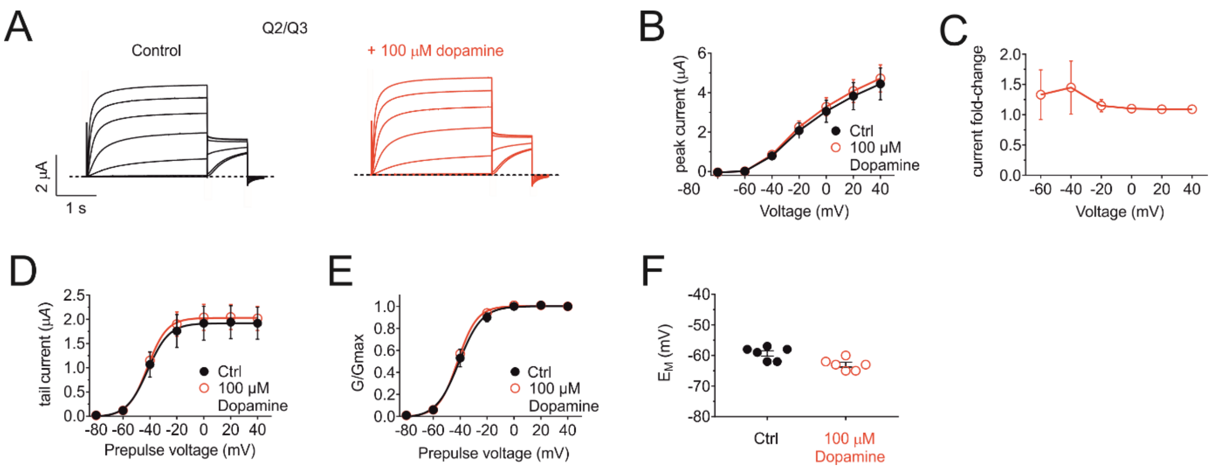

Supplementary Figure 21. KCNQ2/3 channels are insensitive to dopamine

A. Averaged KCNQ2/3 traces in the absence (black) and presence (red) of dopamine (100 μM), *n* = 6.
B. Mean peak current-voltage relationship for recordings as in panel A, *n* = 6. Error bars indicate SEM.
C. Mean current fold-change versus voltage as in panel A, *n* = 6. Error bars indicate SEM.
D. Mean tail current versus prepulse voltage relationships as in panel A, *n* = 6. Error bars indicate SEM.
E. Mean normalized tail current versus prepulse voltage relationships as in panel A, *n* = 6. Error bars indicate SEM.
F. Scatter plot of resting membrane potential (E_M) as in panel A, *n* = 6. Error bars indicate SEM.

**Q2/Q3**	**Normalized tail current $V_{0.5}$ (mV)**	**Non-normalized tail current $V_{0.5}$ (mV)**	**Slope (mV)**
**Ctrl**	-40.8 ± 1.4 (n =6)	-41.6 ± 5.3 (n =6)	8.4 ± 1.5 (n =6)
**100 μM Dopamine**	-41.9 ± 0.6 (n =6)	-41.8 ± 3.6 (n =6)	7.2 ± 0.6 (n =6)

Supplementary Table 21. Summary of Effects of dopamine on KCNQ2/3 channels.

Values indicate mean ± SEM.

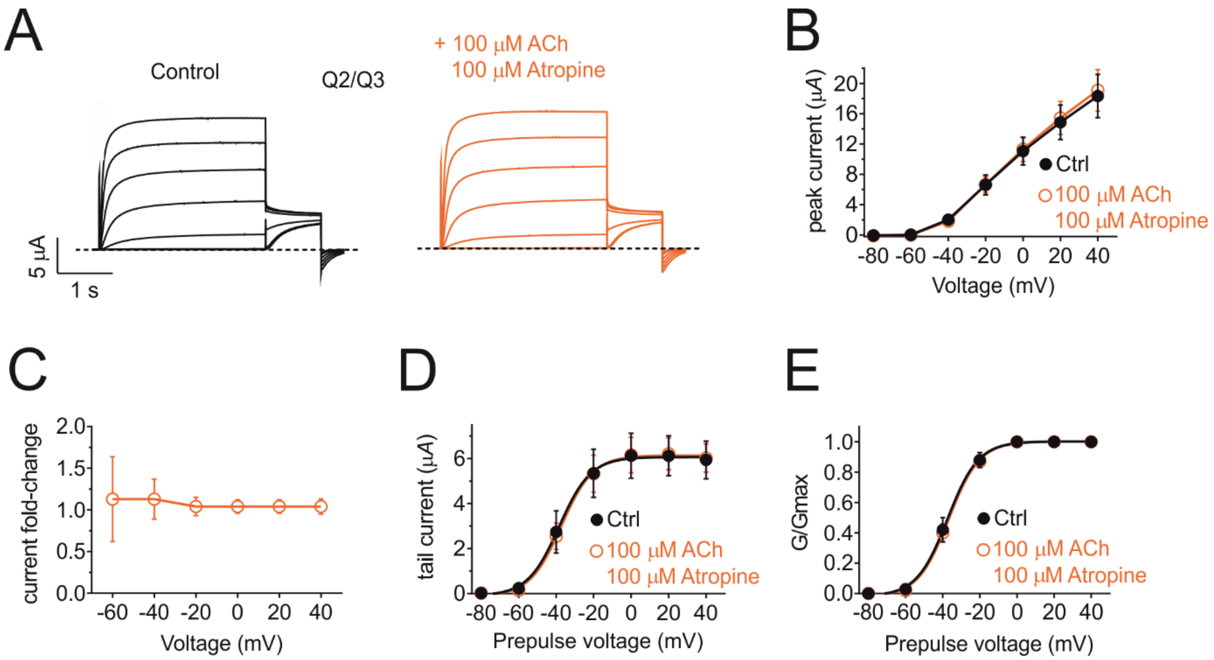

Supplementary Figure 22. Atropine prevents acetylcholine-mediated inhibition of KCNQ2/3 channels

A. Averaged KCNQ2/3 traces in the absence (black) and presence (orange) of Acetylcholine (10 μM) and atropine (100 μM), *n* = 5.
B. Mean peak current-voltage relationship as in panel A, *n* = 5. Error bars indicate SEM.
C. Mean current fold-change versus voltage as in panel A, *n* = 5. Error bars indicate SEM.
D. Mean tail current versus prepulse voltage relationships as in panel A, *n* = 5. Error bars indicate SEM.
E. Mean normalized tail current versus prepulse voltage relationships as in panel A, *n* = 5. Error bars indicate SEM.

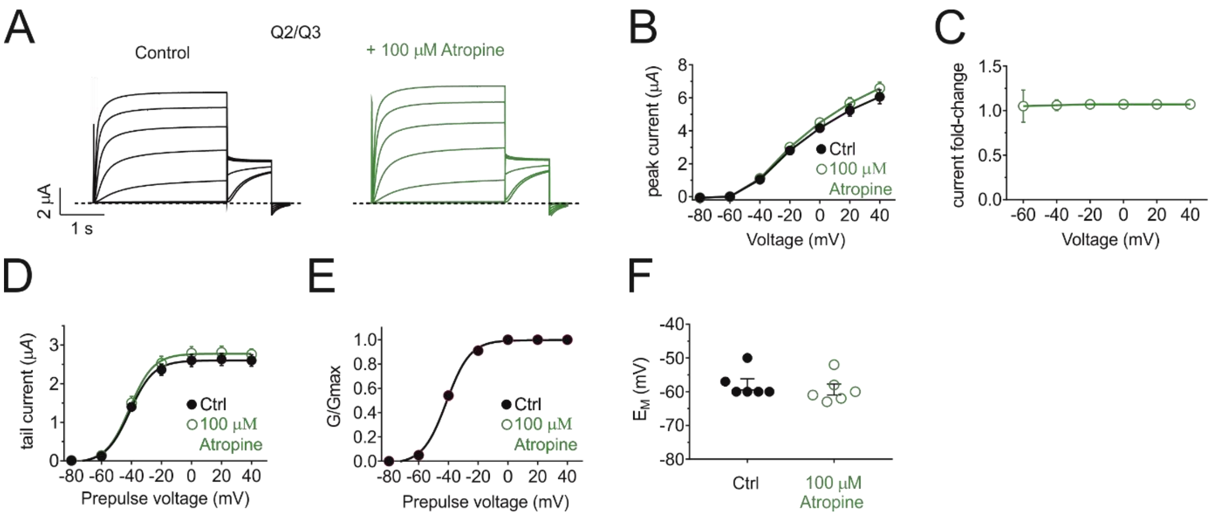

Supplementary Figure 23. Lack of effects of atropine on KCNQ2/3 channels

A. Averaged KCNQ2/3 traces in the absence (black) and presence (green) of atropine (100 µM), *n* = 5.
B. Mean peak current-voltage relationship as in panel A, *n* = 5. Error bars indicate SEM.
C. Mean current fold-change versus voltage as in panel A, *n* = 5. Error bars indicate SEM.
D. Mean tail current versus prepulse voltage relationships as in panel A, *n* = 5. Error bars indicate SEM.
E. Mean normalized tail current versus prepulse voltage relationships as in panel A, *n* = 5. Error bars indicate SEM.
F. Scatter plot of resting membrane potential (E_M) from oocytes as in panel A, *n* = 5. Error bars indicate SEM.

Q2/Q3	Normalized tail current $V_{0.5}$ (mV)	Non-normalized tail current $V_{0.5}$ (mV)	Slope (mV)
**Ctrl**	-41.3 ± 0.4 (n =5)	-41.1 ± 1.8 (n =5)	7.9 ± 0.5 (n =5)
**100 µM Atropine**	-41.3 ± 0.7 (n =5)	-41.2 ± 1.9 (n =5)	7.9 ± 0.8 (n =5)

Supplementary Table 22. Summary of Effects of atropine on KCNQ2/3 channels.

Values indicate mean ± SEM.

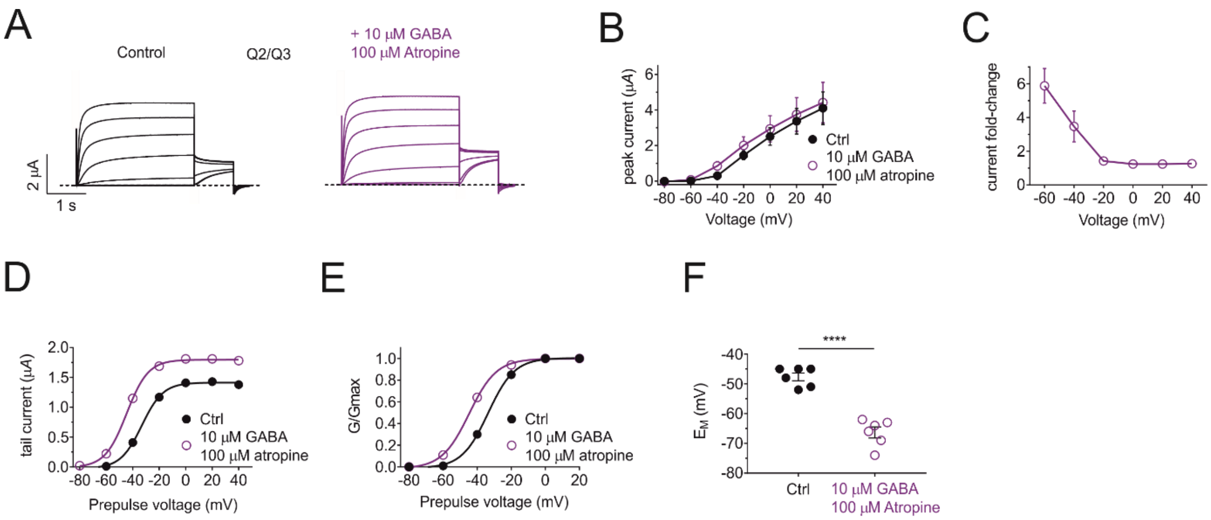

Supplementary Figure 24. Atropine exhibits no effect on GABA sensitivity of KCNQ2/3 channels

A. Averaged KCNQ2/3 traces in the absence (black) and presence (purple) of GABA (10 μM) and atropine (100 μM), *n* = 5.
B. Mean peak current-voltage relationship as in panel A, *n* = 5. Error bars indicate SEM.
C. Mean current fold-change versus voltage as in panel A, *n* = 5. Error bars indicate SEM.
D. Mean tail current versus prepulse voltage relationships as in panel A, *n* = 5. Error bars indicate SEM.
E. Mean normalized tail current versus prepulse voltage relationships as in panel A, *n* = 5. Error bars indicate SEM.
F. Scatter plot of unclamped resting membrane potential (E_M) for oocytes as in panel A, *n* = 5, ****P<0.0001 versus control. Error bars indicate SEM.

Q2/Q3	Normalized tail current $V_{0.5}$ (mV)	Non-normalized tail current $V_{0.5}$ (mV)	Slope (mV)
**Ctrl**	-33.6 ± 0.9 (n =5)	-33.1 ± 4.9 (n =5)	7.7 ± 0.8 (n =5)
**10 μM GABA 100 μM Atropine**	-47.7 ± 1.1 (n =5) ****	-44.6 ± 8.4 (n =5)	8.0 ± 0.9 (n =5)

Supplementary Table 23. Summary of effects of atropine on GABA sensitivity of KCNQ2/3 channels.

Statistics versus same channel in absence of 10 μM GABA and 100 μM atropine: ****p<0.0001. Values indicate mean ± SEM.

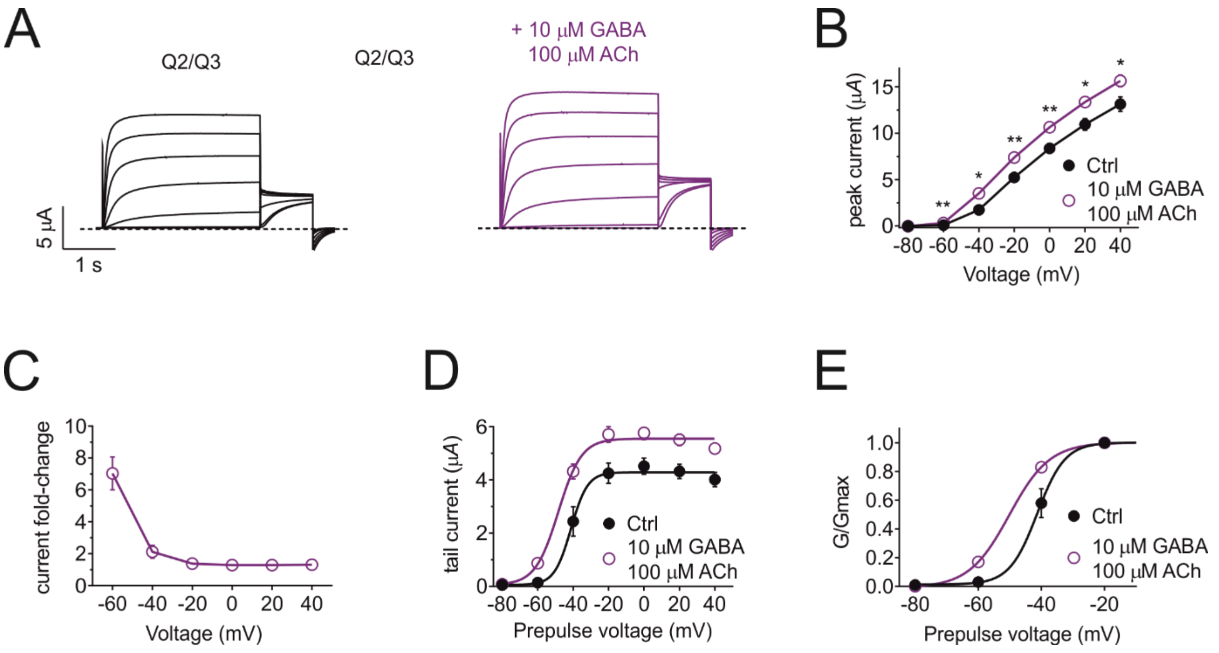

Supplementary Figure 25. GABA overcomes acetylcholine inhibition of KCNQ2/3 channels

A. Averaged KCNQ2/3 traces in the absence (black) and presence (purple) of GABA (10 μM) and ACh (100 μM), *n* = 5.

B. Mean peak current-voltage relationship for recordings as in panel A, *n* = 5. *P<0.05; **P<0.01, versus same-voltage control. Error bars indicate SEM.

C. Mean current fold-change versus voltage as in panel A, *n* = 6. Error bars indicate SEM.

D. Mean tail current versus prepulse voltage relationships as in panel A, *n* = 6. Error bars indicate SEM.

E. Mean normalized tail current versus prepulse voltage relationships as in panel A, *n* = 6. Error bars indicate SEM.

Q2/Q3	Normalized tail current $V_{0.5}$ (mV)	Non-normalized tail current $V_{0.5}$ (mV)	Slope (mV)
**Ctrl**	-41.3 ± 1.3 (n =6)	-41.9 ± 2.1 (n =6)	4.4 ± 3.2 (n =6)
**10 μM GABA 100 μM ACh**	-50.2 ± 0.6 (n =6) ***	-48.3 ± 1.7 (n =6) *	6.3 ± 0.4 (n =6)

Supplementary Table 24. Summary of Effects of acetylcholine on GABA sensitivity of KCNQ2/3 channels.

Statistics versus same channel in absence of 10 μM GABA and 100 μM acetylcholine: ***p=0.0004, *p=0.04. Values indicate mean ± SEM.

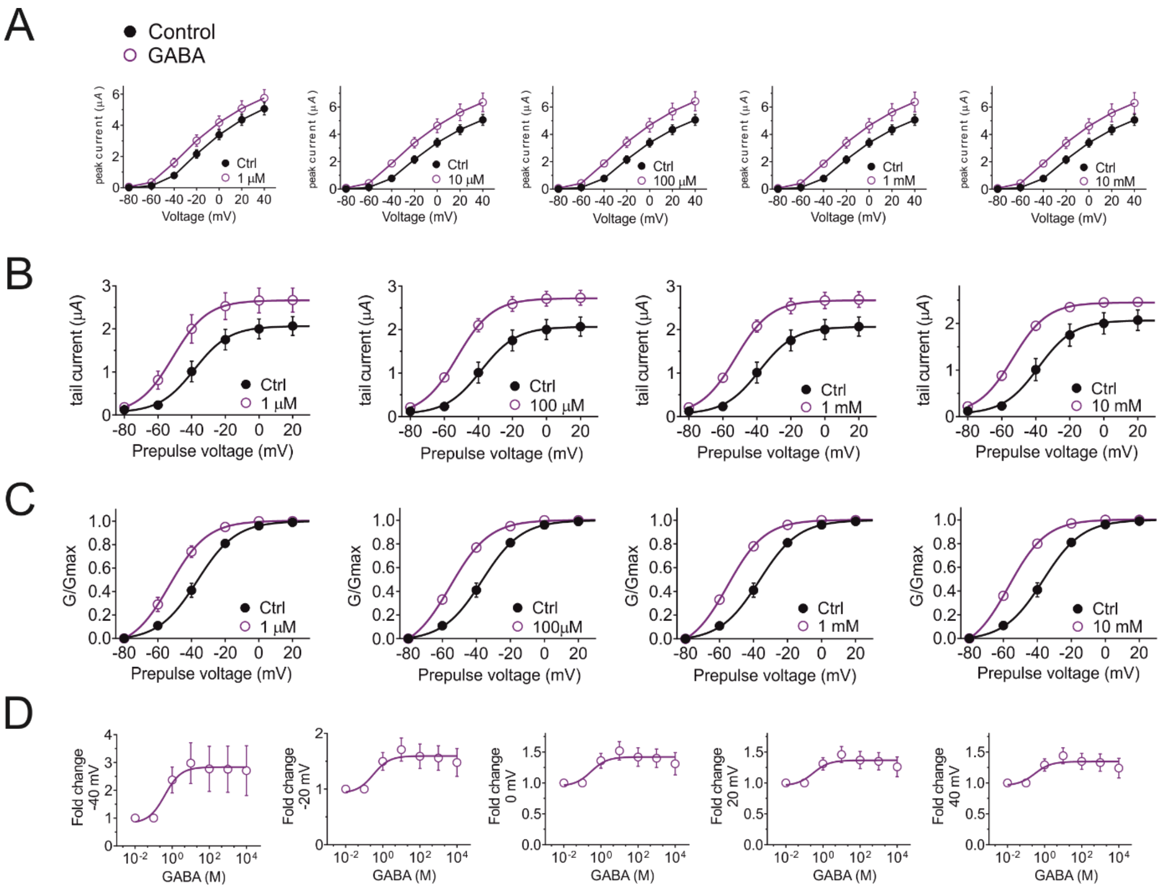

Supplementary Figure 26. GABA effects on KCNQ2/3 after pre-incubation with wortmannin

A. Mean peak current-voltage relationship for KCNQ2/Q3 channels in the absence (black) and presence (purple) of GABA, *n* = 5. Error bars indicate SEM.
B. Mean tail current versus prepulse voltage relationships as in panel A, *n* = 5. Error bars indicate SEM.
C Mean normalized tail current versus prepulse voltage relationships as in panel A, *n* = 5. Error bars indicate SEM.
D. Mean dose response of KCNQ2/3 channels between -40 and +40 mV, *n* = 5. Error bars indicate SEM.

Q2/Q3	Normalized tail current $V_{0.5}$ (mV)	Non-normalized tail current $V_{0.5}$ (mV)	Slope (mV)
**Ctrl**	-34.9 ± 1.4 (n =5)	-34.3 ± 4.6 (n =5)	11.5 ± 1.3 (n =5)
**1 µM GABA**	-52.9 ± 1.9 (n =5) ***	-54.7 ± 6.4 (n =5)	11.2 ± 1.6 (n =5)
**10 µM GABA**	-57.6 ± 1.3 (n =5) ****	-57.8 ± 5.4 (n =5)	10.8 ± 0.9 (n =5)
**100 µM GABA**	-55.4 ± 0.9 (n =5) ****	-55.4 ± 3.3 (n =5) *	11.5 ± 0.7 (n =5)
**1 mM GABA**	-55.1 ± 0.8 (n =5) ****	-55.5 ± 3.5 (n =5) *	10.9 ± 0.6 (n =5)
**10 mM GABA**	-56.8 ± 0.9 (n =5) ****	-56.9 ± 1.6 (n =5) *	10.9 ± 0.7 (n =5)

Supplementary Table 25. Summary of Effects of wortmannin on GABA sensitivity of KCNQ2/3 channels.

Statistics versus same channel in absence of GABA: ****p<0.0001, ***p=0.002, *p=0.01. Values indicate mean ± SEM.

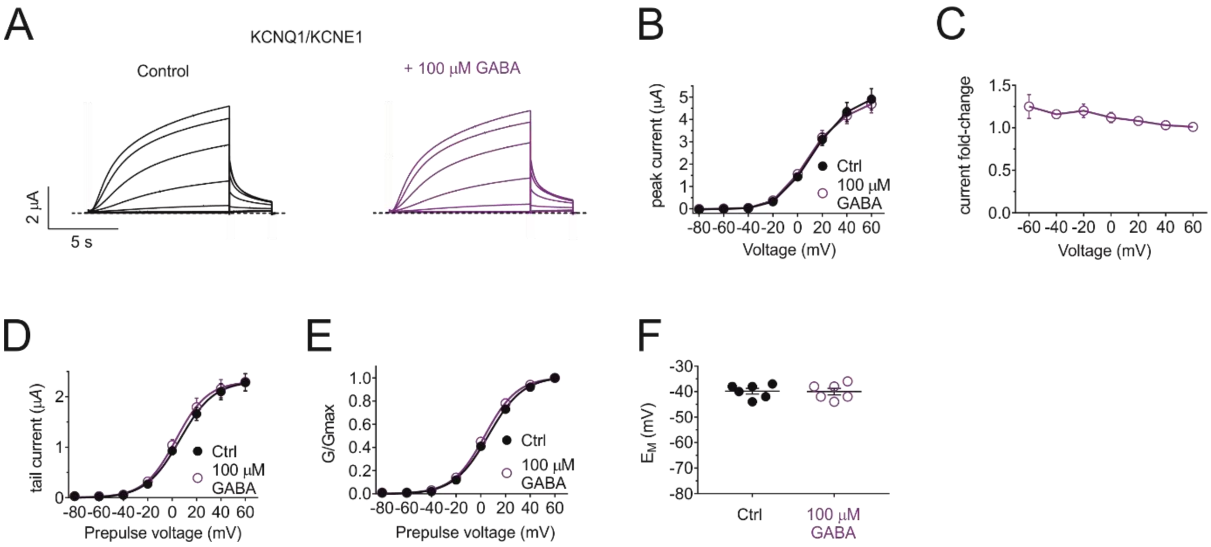

Supplementary Figure 27. Effects of GABA on KCNQ1-KCNE1 channels

A. Averaged KCNQ1/KCNE1 traces in the absence (black) and presence (purple) of GABA (100 μM), *n* = 6.
B. Mean peak current-voltage relationship as in panel A, *n* = 6. Error bars indicate SEM.
C. Mean current fold-change versus voltage as in panel A, *n* = 6. Error bars indicate SEM.
D. Mean tail current versus prepulse voltage relationships as in panel A, *n* = 6. Error bars indicate SEM.
E. Mean normalized tail current versus prepulse voltage relationships as in panel A, *n* = 6. Error bars indicate SEM.
F. Scatter plot of resting membrane potential (E_M) as in panel A, *n* = 6. Error bars indicate SEM.

**Q1/E1**	**Normalized tail current $V_{0.5}$ (mV)**	**Non-normalized tail current $V_{0.5}$ (mV)**	**Slope (mV)**
**Ctrl**	6.1 ± 0.8 (n =6)	6.3 ± 2.6 (n =6)	14 ± 0.7 (n =6)
**100 μM GABA**	3.3 ± 1.2 (n =6)	3.1 ± 2.7 (n =6)	13.2 ± 0.9 (n =6)

Supplementary Table 26. Summary of Effects of GABA on KCNQ1/KCNE1 channels.

Values indicate mean ± SEM.

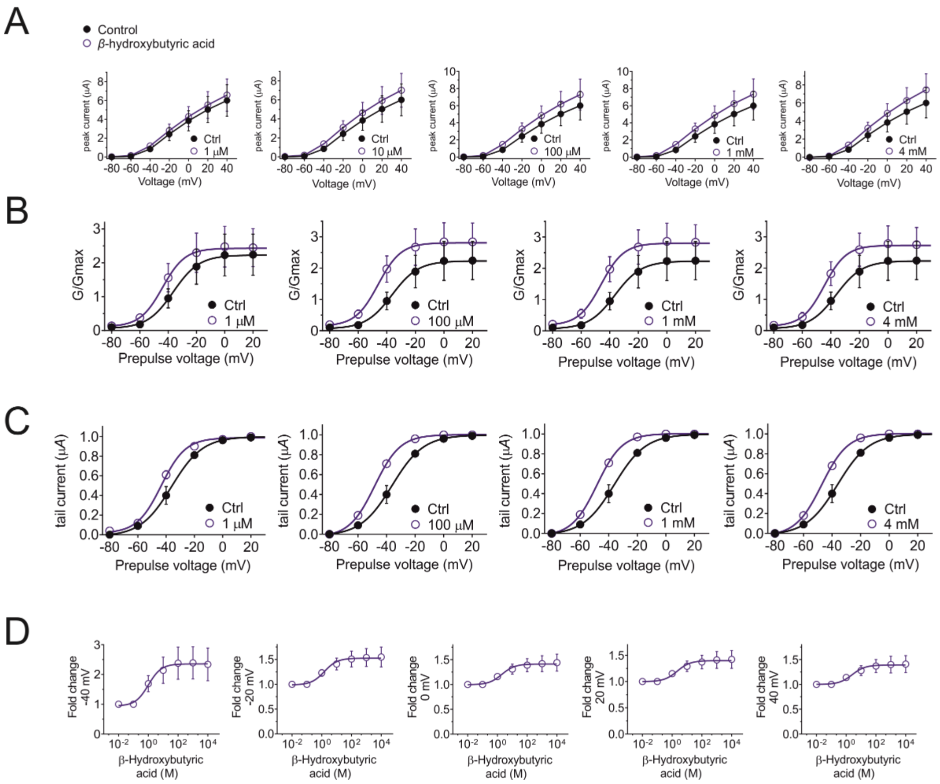

Supplementary Figure 28. Effects of β-Hydroxybutyric acid on KCNQ2/3 channels

A. Mean peak current-voltage relationship for KCNQ2/3 channels in the absence (black) and presence (blue) of *β*-Hydroxybutyric acid, *n* = 5. Error bars indicate SEM.

B. Mean tail current versus prepulse voltage relationships as in panel A, *n* = 5. Error bars indicate SEM.

C. Mean normalized tail current versus prepulse voltage relationships as in panel A, *n* = 5. Error bars indicate SEM.

D. Mean dose response of KCNQ2/3 channels between -40 and +40 mV, *n* = 5. Error bars indicate SEM.

**Q2/Q3**	**Normalized tail current $V_{0.5}$ (mV)**	**Non-normalized tail current $V_{0.5}$ (mV)**	**Slope (mV)**
**Ctrl**	-36.1 ± 1.8 (n =5)	-36.4 ± 8.7 (n =5)	6.2 ± 0.8 (n =5)
**1 µM *β*-Hydrox**	-46.1 ± 0.6 (n =5) **	-43.9 ± 8.2 (n =5)	5.9 ± 0.4 (n =5)
**10 µM *β*-Hydrox**	-48.1 ± 0.4 (n =5) **	-45.5 ± 7.9 (n =5)	5.9 ± 0.4 (n =5)
**100 µM *β*-Hydrox**	-50.2 ± 0.3 (n =5) **	-46.0 ± 7.9 (n =5)	5.9 ± 0.3 (n =5)
**1 mM *β*-Hydrox**	-46.5 ± 0.9 (n =5) **	-46.0 ± 7.4 (n =5)	5.7 ± 0.6 (n =5)
**4 mM *β*-Hydrox**	-44.9 ± 0.8 (n =5) **	-44.7 ± 7.1 (n =5)	4.9 ± 0.6 (n =5)

Supplementary Table 27. Summary of Effects of *β*-Hydroxybutyric acid on KCNQ2/3 channels.

Statistics versus same channel in absence of *β*-Hydroxybutyric acid: **p=0.001. Values indicate mean ± SEM.

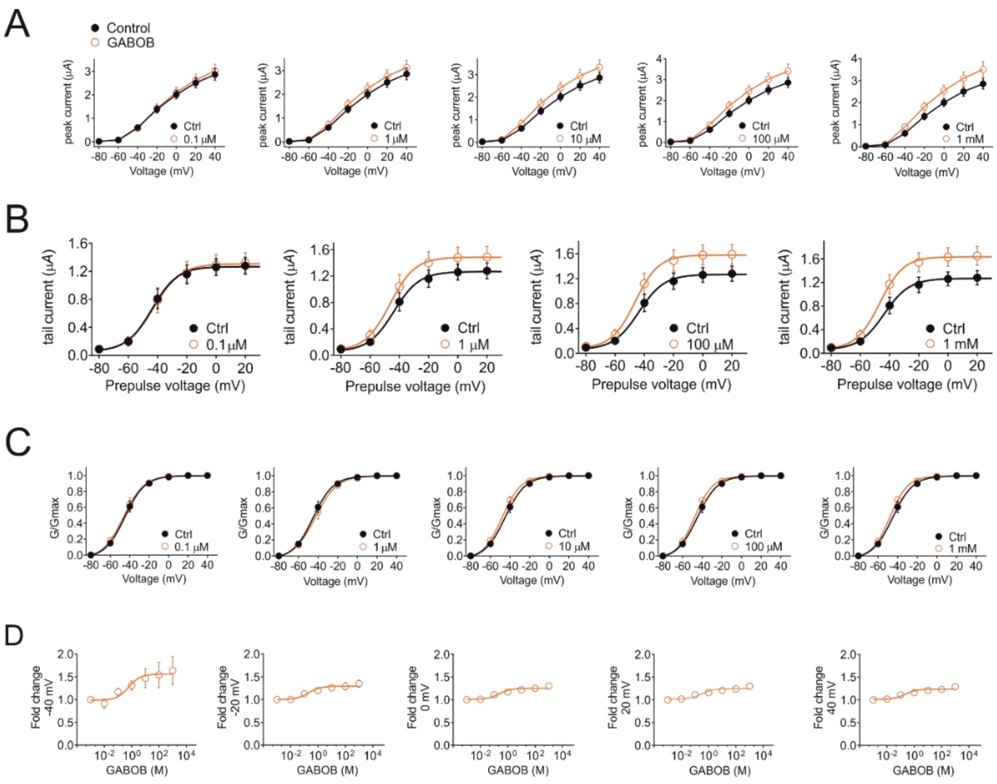

Supplementary Figure 29. Effects of GABOB on KCNQ2/3 channels

A. Mean peak current-voltage relationship for KCNQ2/3 channels in the absence (black) and presence (orange) of GABOB, *n* = 6. Error bars indicate SEM.

B. Mean tail current versus prepulse voltage relationships as in panel A, *n* = 6. Error bars indicate SEM.

C. Mean normalized tail current versus prepulse voltage relationships as in panel A, *n* = 6. Error bars indicate SEM.

D. Mean dose response of KCNQ2/3 channels between -40 and +40 mV, *n* = 6. Error bars indicate SEM.

Q2/Q3	Normalized tail current $V_{0.5}$ (mV)	Non-normalized tail current $V_{0.5}$ (mV)	Slope (mV)
**Ctrl**	-44.9 ± 1.5 (n =6)	-43.8 ± 3.9 (n =6)	10.3 ± 1.3 (n =6)
**0.01 µM GABOB**	-42.8 ± 2.0 (n =6)	-42.7 ± 5.0 (n =6)	11.1 ± 1.8 (n =6)
**0.1 µM GABOB**	-46.6 ± 1.9 (n =6)	-45.8 ± 5.1 (n =6)	10.6 ± 1.6 (n =6)
**1 µM GABOB**	-47.1 ± 1.4 (n =6)	-46.4 ± 4.7 (n =6)	10.1 ± 1.2 (n =6)
**10 µM GABOB**	-48.1 ± 1.2 (n =6)	-46.8 ± 4.5 (n =6)	9.6 ± 0.9 (n =6)
**100 µM GABOB**	-47.8 ± 0.9 (n =6)	-46.3 ± 4.2 (n =6)	9.3 ± 0.8 (n =6)
**1 mM GABOB**	-48.0 ± 0.9 (n =6)	-46.7 ± 4.0 (n =6)	9.2 ± 0.7 (n =6)

Supplementary Table 28. Summary of Effects of GABOB on KCNQ2/3 channels.

Values indicate mean ± SEM.

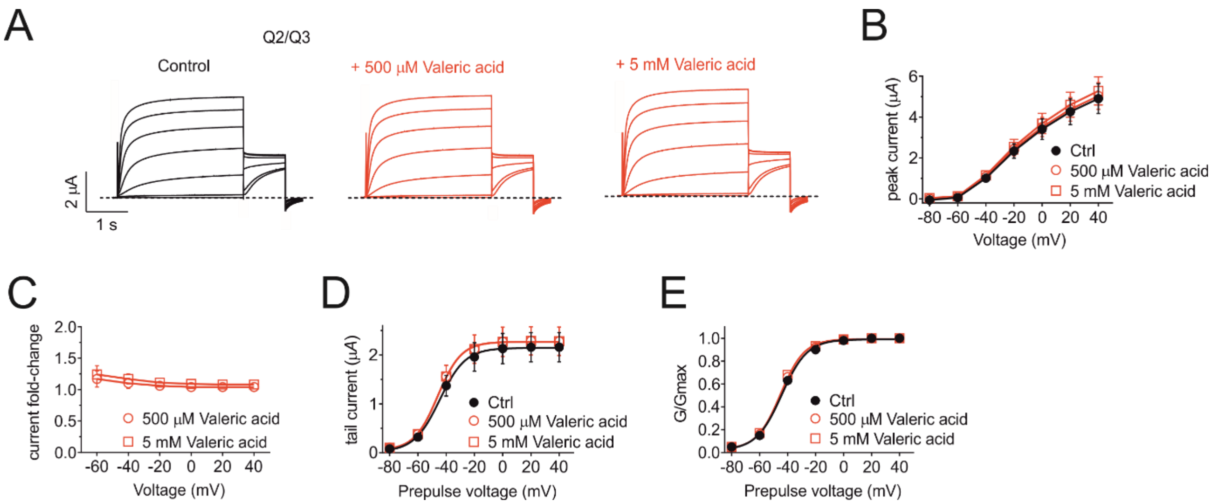

Supplementary Figure 30. Lack of effects of valeric acid on KCNQ2/3 channels

A. Mean peak current-voltage relationship for KCNQ2/3 channels in the absence (black) and presence (red) of valeric acid (500 μM; middle, 5 mM; right), *n* = 6.

B. Mean peak current-voltage relationship as in panel A, *n* = 6. Error bars indicate SEM.

C. Mean current fold-change versus voltage as in panel A, *n* = 6. Error bars indicate SEM.

D. Mean tail current versus prepulse voltage relationships as in panel A, *n* = 6. Error bars indicate SEM.

E. Mean normalized tail current versus prepulse voltage relationships as in panel A, *n* = 6. Error bars indicate SEM.

Q2/Q3	Normalized tail current $V_{0.5}$ (mV)	Non-normalized tail current $V_{0.5}$ (mV)	Slope (mV)
**Ctrl**	-44.2 ± 0.6 (n =6)	-44.5 ± 5.4 (n =6)	9.1 ± 0.5 (n =6)
**500 µM Valeric acid**	-45.1 ± 0.6 (n =6)	-45.6 ± 4.9 (n =6)	8.5 ± 0.6 (n =6)
**500 mM Valeric acid**	-45.1 ± 0.6 (n =6)	-45.9 ± 4.9 (n =6)	8.5 ± 0.6 (n =6)

Supplementary Table 29. Summary of Effects of valeric acid on KCNQ2/3 channels.

Values indicate mean ± SEM.

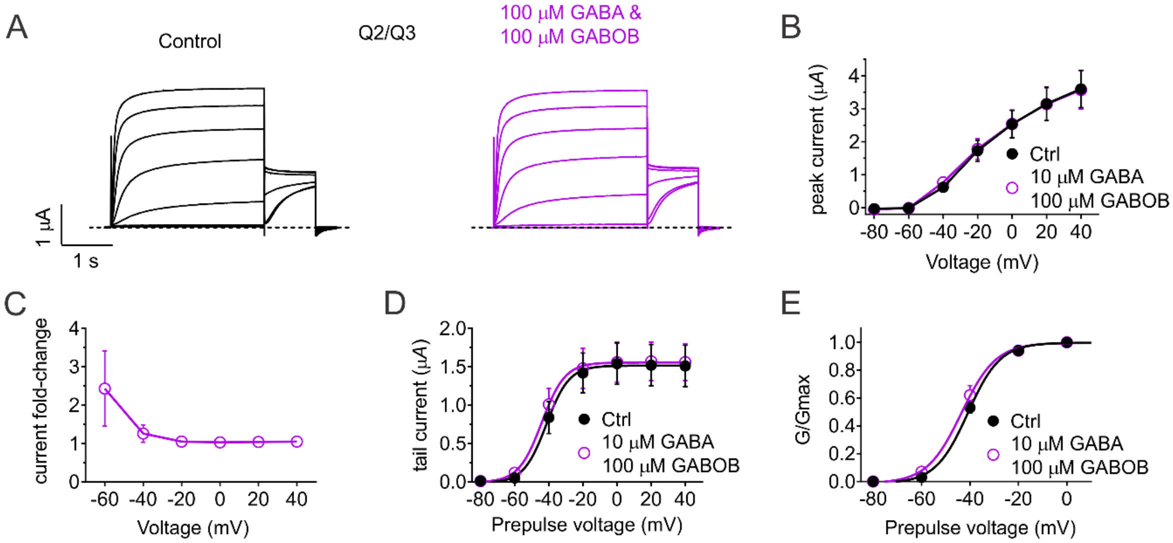

Supplementary Figure 31. GABOB reduces GABA sensitivity in KCNQ2/3 channels

A. Averaged KCNQ2/3 traces in the absence (black) and presence (purple) of GABA (10 μM) and GABOB (100 μM), *n* = 4.
B. Mean peak current-voltage relationship for recordings as in panel A, *n* = 4. Error bars indicate SEM.
C. Mean current fold-change versus voltage as in panel A, *n* = 4. Error bars indicate SEM.
D. Mean tail current versus prepulse voltage relationships as in panel A, *n* = 4. Error bars indicate SEM.
E. Mean normalized tail current versus prepulse voltage relationships as in panel A, *n* = 4. Error bars indicate SEM.

**Q2/Q3**	**Normalized tail current $V_{0.5}$ (mV)**	**Non-normalized tail current $V_{0.5}$ (mV)**	**Slope (mV)**
**Ctrl**	-48.1 ± 0.7 (n =4)	-47.0 ± 2.4 (n =4)	6.7 ± 0.5 (n =4)
**10 μM GABA 100 μM GABOB**	-51.0 ± 0.7 (n =4)	-49.6 ± 2.0 (n =4)	7.2 ± 0.4 (n =4)

Supplementary Table 30. Summary of effects of GABOB on GABA sensitivity of KCNQ2/3 channels.

Values indicate mean ± SEM.

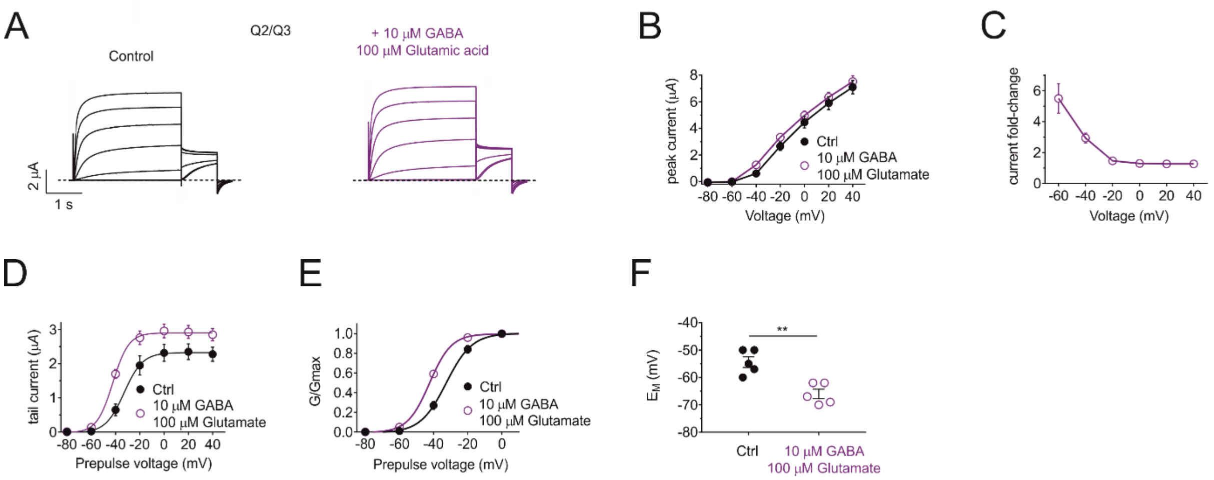

Supplementary Figure 32. Glutamate has no effect on GABA activation of KCNQ2/3 channels
A. Averaged KCNQ2/3 traces in the absence (black) and presence (purple) of GABA (10 μM) and glutamate (100 μM), *n* = 5.
B. Mean peak current-voltage relationship as in panel A, *n* = 5. Error bars indicate SEM.
C. Mean current fold-change versus voltage as in panel A, *n* = 5. Error bars indicate SEM.
D. Mean tail current versus prepulse voltage relationships as in panel A, *n* = 5. Error bars indicate SEM.
E. Mean normalized tail current versus prepulse voltage relationships as in panel A, *n* = 5. Error bars indicate SEM.
F. Scatter plot of resting membrane potential (E_M) as in panel A, *n* = 5, **P<0.002 versus control. Error bars indicate SEM.

**Q2/Q3**	**Normalized tail current $V_{0.5}$ (mV)**	**Non-normalized tail current $V_{0.5}$ (mV)**	**Slope (mV)**
**Ctrl**	-32.5 ± 0.8 (n =5)	-32.9 ± 3.1 (n =5)	7.5 ± 0.6 (n =5)
**10 μM GABA 100 μM Glutamate**	-42.4 ± 0.2 (n =5) ***	-42.4 ± 2.2 (n =5) *	6.4 ± 0.2 (n =5)

Supplementary Table 31. Summary of Effects of glutamate on GABA sensitivity of KCNQ2/3 channels.

Statistics versus same channel in absence of 10 μM GABA and 100 μM glutamate: ***p=0.0001, *p=0.03. Values indicate mean ± SEM.
